# Canonical and noncanonical forms of G4 DNA at cluster III of the *BCL6* breakpoint region could lead to chromosomal translocation in DLBCL

**DOI:** 10.1016/j.jbc.2026.111347

**Published:** 2026-03-09

**Authors:** Saniya M. Javadekar, Sayak Das, Sujatha M. Hanumegowda, Susmita Kumari, Bibha Choudhary, Sathees C. Raghavan

**Affiliations:** 1 Department of Biochemistry, Indian Institute of Science, Bangalore, India; 2 Institute of Bioinformatics and Applied Biotechnology, Electronics City, Bangalore, India

**Keywords:** double-strand breaks, chromosomal translocation;, genome stability, non-B DNA, G4 DNA, G-quadruplex DNA

## Abstract

Cancer arises from the accumulation of genetic alterations, including chromosomal translocations and deletions. Faulty repair of DNA double-strand breaks can give rise to such chromosomal rearrangements. In this study, we focus on diverse translocations that share a common partner, *BCL6* on chromosome 3, which are implicated in diffuse large B-cell lymphoma (DLBCL). Analysis of patient breakpoints identified several breakpoint clusters within *BCL6*, of which Cluster III is the focus of this work. Here, we investigate the role of non-B DNA structures in imparting chromosomal fragility. In silico analyses, gel shift assays, and circular dichroism confirmed G-quadruplex (G4) formation at *BCL6* Cluster III. Mutation studies revealed multiple G4 conformations utilizing distinct G-stretches, including GNG motifs. Disrupting G4-forming sequences in this region enhanced plasmid propagation in *E. coli*, indicating structure-dependent replication stalling. Sodium bisulfite modification assays detected single-stranded character here, both in plasmids and chromosomal DNA, suggesting additional fragility hotspots within Cluster III. *Ex vivo* assays showed that the G4 structure blocks transcription as a roadblock. Together, these data demonstrate that G4 folding in *BCL6* Cluster III generates partially single-stranded regions, rendering the locus prone to breakage and translocation.

Diffuse large B-cell lymphoma (DLBCL), accounting for ∼40% of non-Hodgkin lymphomas, frequently harbors chromosomal rearrangements involving *BCL6*, *MYC*, and *BCL2* (1, 2, 3). *BCL6*, a proto-oncogene at chromosome 3q27, spans 26 kb across 10 exons, with the protein synthesis signal in exon 3 (4). It encodes BCL6, a sequence-specific transcription repressor essential for germinal center (GC) B-cell formation, survival, and maintenance amid high rates of somatic hypermutation (4, 5). *BCL6* expression is tightly regulated during B-cell differentiation (6, 7), repressing genes that enable GC exit and plasma cell differentiation. This regulation renders B cells resistant to uncontrolled proliferation and the mutagenic effects of DNA-editing enzymes required for immunoglobulin affinity maturation (8). By controlling DNA damage sensing and cell proliferation checkpoints (9), BCL6 maintains genomic stability. Disruption of its normal downregulation impairs target gene repression, promotes genetic instability, and drives B-cell malignant transformation (10).

A prime cause of disruption in BCL6 expression is a chromosomal translocation associated with *BCL6*, which is reported in almost 40% of cases with diffuse large B-cell lymphomas (DLBCL) and 6 to 15% of cases with follicle lymphomas (FL) (11, 12, 13, 14). The chromosomal translocations associated with DLBCL samples involving the *BCL6* locus have almost 49 immunoglobulin (Ig) and non-Ig genes acting as the translocation partners (Atlas Genetics Oncology; https://atlasgeneticsoncology.org) (Fig. 1*A*). Most *BCL6* rearrangements involve promoter substitutions. The precise mechanism underlying *BCL6* fragility remains largely unknown, although recent reports suggest that non-B DNA structures at Cluster II contribute to this genomic instability (15).

**Figure 1:**
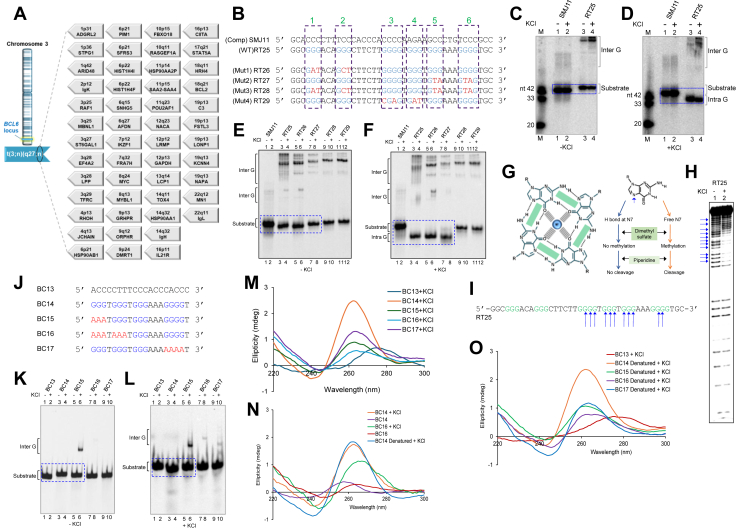
**Sequence and structure analysis of *BCL6* cluster III within DLBCL patient breakpoint region at the 5′UTR of the gene.***A*, schematic illustration of chromosome three harboring *BCL6* locus at q27 (indicated in *blue*) and its multiple translocation partners (n; indicated by band positions and locus name) in the *grey boxes* alongside chromosome 3. *B*, Wild-type sequence of G4 motif (RT25) from *BCL6* Cluster III along with complementary and mutated counterparts used for the study are shown. G stretches are indicated in *blue*, and mutations are in *red*. Numbered boxes indicate the relevant guanine or cytosine stretches in the sequences. *C* and *D*, electrophoretic mobility shift assays were performed in the absence (*C*) or presence (*D*) of 100 mM KCl using the complementary C-rich strand SMJ11 (lanes 1–2) and the wild-type oligomer RT25 (lanes 3–4). Mobility shifts corresponding to intramolecular structures were indicated by *blue boxes*, while intermolecular species were labeled. The substrates were resolved on 12% native polyacrylamide gels in the presence or absence of 25 mM potassium chloride. A molecular weight marker composed of three oligomers of defined lengths (20, 33, and 42 nt) was employed to facilitate accurate comparison of band migration. The - and +, at the *bottom*, in the *left* and *right* gel respectively, indicates the absence and presence of KCl in the gel and buffer. *E and F*, electromobility shift assay in absence (*E*) or presence (*F*) of 100 mM KCl for wild type oligomer RT25 (lanes 3–4), complementary C-strand SMJ11 (lanes 1–2) and mutant sequences RT26 to 29 (lanes 5–12). Shifts in mobility of intramolecular structures are boxed in *blue* while intermolecular structures are labelled. The substrates were resolved on 12% native polyacrylamide gels with or without 25 mM potassium chloride. *G*, schematic showing involvement of guanines in G4-structure (tetrad) formation (Hoogsteen base-pairing is indicated by green box between guanines and is stabilized by cations-indicated by M^+^) in absence or presence of KCl, followed by dimethyl sulfate treatment and further cleavage by piperidine. The reaction products were resolved on 18% denaturing PAGE. *H*, denaturing PAGE showing piperidine-cleaved guanine residues in RT25 in absence (lane 1) or presence of 100 mM KCl (lane 2). Alongside sequence of wild type *BCL6* with guanines marked in green. The guanines that show DMS protection are marked by *blue arrows*. *I*, Wild-type *BCL6* sequence with guanines marked in *green*, and the protected guanines in presence of KCl, are marked with blue arrows. *J*, oligomeric sequence of shorter G-rich strand, the complementary strand, and the mutated counterparts derived from *BCL6* Cluster III region. G stretches are shown in *blue* and mutations in *red*. *K and L*, radiolabeled wild type strand BC14, complementary C-strand BC13 and mutants BC13 to 17 were incubated in TE (pH 8.0) in absence (*K*) and presence (*L*) of 100 mM KCl at 37 °C for 1 h and resolved on 15% native PAGE. Wild-type oligomer BC14 (lanes 3–4) represents G-strand while BC13 (lanes 1–2) represents C-strand and other mutated sequences are represented as BC15 to 17 (lanes 5–10). Mobility shifts of intramolecular species are boxed in *blue*. Intermolecular structures are labelled. *M–O*, circular dichroism spectra were recorded for different substrates in presence of 100 mM KCl. The substrates used were wild type G strand (BC 14), its complementary C strand (BC13) and the mutants (BC15, BC16, BC17) (M). CD spectra of heat denatured substrates upon renaturation in presence of KCl were recorded (N-O). All spectra were recorded at room temperature from 220 to 300 nm, at a scan speed of 50 nm/min, and are plotted as function of wavelength (nm) on X-axis and ellipticity (mdeg) on Y-axis.

Structurally, the highly conserved first intron and first non-coding exon of *BCL6* contain regulatory elements at their 5′ extremity. This region is also characterized by frequent genetic alterations in lymphomas (16). To better understand the potential causes of this local fragility, DLBCL patients with DLBCL sequences were assessed for chromosomal translocation breakpoints from several published studies (17, 18, 19, 20, 21, 22, 23, 24, 25, 26, 27, 28) and were plotted on genomic *BCL6* gene sequence, indicating breakpoint clustering within a 4 kb region (major translocation cluster) of 5′UTR of *BCL6.* The pattern of breakpoint distribution showed three peaks (designated as cluster I, cluster II, and cluster III), where most of the breakpoints were clustered (Fig. S1). In this study, we focused on Cluster III, which harbors a high density of patient breakpoints. Through complementary biochemical and biophysical approaches, we demonstrated the formation of multiple G-quadruplex (G4) DNA structures at the *BCL6* Cluster III breakpoint region. Bisulfite modification assays revealed single-strandedness on the strand complementary to the G4-forming sequence, both in plasmid DNA and in genomic contexts. These G4 structures blocked DNA replication and transcription *in vitro* and within cells. Collectively, our data indicate that G4 formation at *BCL6* Cluster III imparts genomic fragility, promoting translocation events.

## Results

### G4 DNA structure is formed at the DNA sequence from *BCL6*-cluster III and the identification of guanine residues associated with it

To assess non-B DNA structure formation in the patient breakpoint region at *BCL6* Cluster III (Fig. S4, *A* and *B*), oligonucleotides corresponding to the predicted G4 motif were synthesized, along with mutants designed to disrupt guanine stretches (Fig. 1*B*). A wild-type 40 nt G-rich oligomer (RT25) with six guanine stretches and its complementary C-rich strand (SMJ11) served as controls. Mutants included RT26 (two 5′ guanine stretches mutated), RT27 (two 3′ stretches mutated), RT28 (5′ and 3′ stretches mutated, central stretches intact), and RT29 (central stretches mutated, outer stretches intact).

Gel mobility shift assays were performed using radiolabeled oligomers resolved on native polyacrylamide gels in the presence or absence of KCl in both gel and running buffer. A molecular weight marker (20, 33, 42 nt oligonucleotides) ensured accurate mobility interpretation (Fig. 1, *C* and *D*). In the absence of KCl, all oligomers migrated according to their expected size, with RT25 comigrating with SMJ11 (Fig. 1*C*). Addition of KCl induced faster migration of RT25 relative to size markers, consistent with potassium-dependent G-quadruplex compaction (Fig. 1*D*). Mutant analysis confirmed structure-specific mobility shifts (Fig. 1, *E* and *F*). In KCl, wild-type RT25 and partial mutants RT26/RT27 migrated faster than SMJ11 (Fig. 1*E*, lanes 3–8; 1F, lanes 1–2), indicating intramolecular G-quadruplex formation. Full mutants RT28/RT29 showed no such shifts regardless of KCl (Fig. 1*E*, lanes 9–12). Higher molecular weight bands observed across all G-rich sequences (RT25–29) suggested intermolecular G-quadruplexes (Fig. 1, *E* and *F*).

To understand precise base pairing during G4 DNA formation, the wild-type oligomer (RT25) was subjected to the chemical probing assay, namely, DMS (dimethyl sulfate) protection assay in the presence and absence of KCl (Fig. 1, *G*–*I*). Results showed that while all guanines showed comparable reactivity, both in the absence of KCl (Fig. 1*H*, lane 1), the cleavage efficacy was significantly decreased at specific guanine residues, which were involved in the structure formation (Fig. 1*H*, lane 2, indicated by arrows). This result revealed that these guanine residues were engaged in Hoogsteen base pairing during the intramolecular G4 DNA formation (Fig. 1*I*, indicated by arrows). Hence, our results identify the residues within the G-rich strand of the *BCL6* Cluster III region that form the G-quadruplex DNA structure.

Further, shorter G-rich oligomeric DNA sequences (19 nt long) were designed from the 3′ end of RT25, featuring at least four stretches of three guanines, along with G sequence mutants (Fig. 1*J*). Results showed that in the presence of KCl, the wild-type G-rich substrate (BC14) migrated faster than the C-rich strand (BC13) (Fig. 1*K*, lanes 3–4; Fig. 1*L*, lanes 3–4), suggesting the formation of potassium-dependent intramolecular G-quadruplex structures. Mutant sequences BC15, BC16, and BC17 (mutations in red) formed intermolecular but not intramolecular species of G-quadruplex in the presence of KCl (Fig. 1*L*, lanes 5–10). To characterize the formation of these non-B DNA structures, circular dichroism (CD) studies were performed. CD spectra of *BCL6* cluster III shorter oligomers exhibited a characteristic positive peak at 265 nm and a dip at 240 nm, indicative of a parallel G-quadruplex on the G-strand (BC14). G-rich sequences (BC14, BC15, BC16, and BC17) were then heat-denatured and renatured in the presence of Potassium (Fig. 1, M and *O*). This allowed for the complete recovery of the G-quadruplex structure in all G-rich sequences, as evident from the spectra (Fig. 1*O*). Mutant sequences (BC15, 16, 17) exhibited short peaks at 265 nm, which may have been contributed by a minor subpopulation of molecules forming inter-G quadruplexes, as observed in the gel shift assays (Fig. 1*M*). Complementary strand C-rich sequence (BC13) showed a positive peak at ∼280 nm and a trough at 240 nm, which was like that of normal single-strand DNA (Fig. 1, *M* and *O*). Taken together, these results demonstrate that *BCL6* Cluster III can fold into a G-quadruplex DNA structure that is stabilized by K^+^ ions.

### Replication through *BCL6* cluster III results in a replication block, which is dependent on potassium and TMPyP4

**Figure 2:**
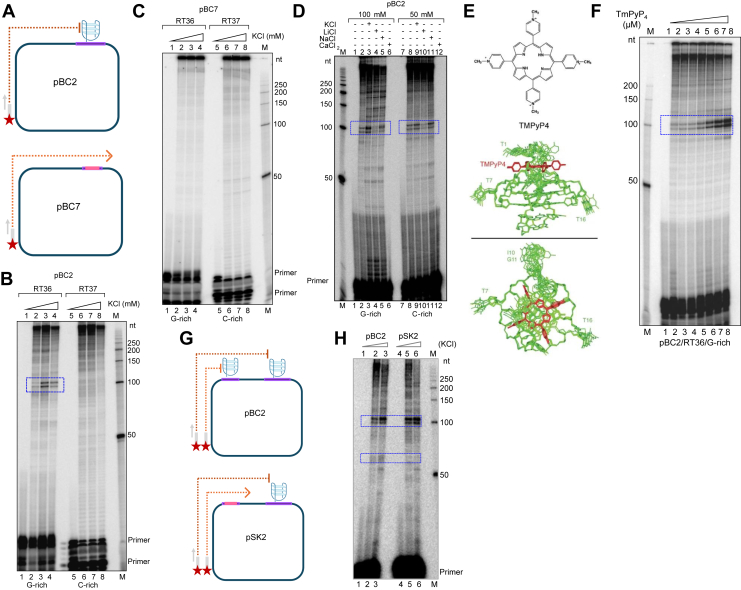
**Primer extension across the G-rich and C-rich regions of *BCL6* Cluster III.***A*, schematic representation of plasmids pBC2 (wild type) harboring G-quadruplex structure and pBC7 (mutant) without any structure forming sequence, were subjected to polymerase extension using radiolabeled primers RT36 and RT37. *B* and *C*, primers RT36 and RT37 were used for extension reactions in pBC2 in the presence of increasing KCl concentration (20, 100, 150 mM) (*B*). Lanes one and five indicate primer without template. Pause sites in G-rich strand are boxed in *blue*. No pauses are observed in the C-rich strand. Primers RT36 and RT37 were used for extension reactions in mutant pBC7 in increasing KCl concentration (20, 100, 150 mM) (C). Lanes one and five are primer without template. "M" denotes the radiolabeled 50 bp ladder and the molecular sizes are marked. *D*, primer extension with RT36 on pBC2 was performed in presence of 100 and 50 mM KCl (lanes 3, 9), LiCl (lanes 4, 10), NaCl (lanes 5, 11), and CaCl_2_ (lanes 6, 12). The pause sites in G-rich region are boxed in *blue*. Lanes one and seven are primer RT36 without template. “M” denotes the radiolabeled 50 bp ladder and the molecular sizes are marked. *E*, structure of G4-stabilizer, TmPyP4 [5,10,15,20-Tetrakis-(N-methyl-4-pyridyl) porphine], a known G4-intercalator; side and top view of interaction of TmPyP4 (shown in red) with the plates of Pu24I G4-structure (shown in *green*; adapted from *Nature Chemical Biology, 1, 167–173, 2005.) F*, primer extension reactions were performed with radiolabeled primer RT36 on pBC2 at increasing concentrations of TmPyP4 (0.05, 0.1, 0.2, 0.5, 1.0, 2.0 μM). Pause sites formed in G-rich strand are boxed in *blue*. Lane one is a primer without template. “M” denotes the radiolabeled 50 bp ladder and the molecular sizes are marked. *G*, schematic representation of plasmids pBC2 (wild type), containing G-quadruplex forming motifs, and pSK2 (mutant), in which one of the G-stretch was mutated. Both plasmids were subjected to polymerase extension assays using radiolabeled primer RT36. *H*, primer extension analysis comparing the wild-type *BCL6* Cluster III construct (pBC2) and the G4-defective mutant construct (pSK2). Reactions were performed in the presence of increasing KCl concentrations (20 and 100 mM), and extension products were resolved on a denaturing polyacrylamide gel. "M" denotes the radiolabeled 50 bp ladder; lanes one and four indicate primer without template. The prominent replication pause site observed in the wild-type construct (highlighted by the *blue box*) was markedly reduced in the pSK2 mutant, indicating loss of replication pausing upon disruption of the G-quadruplex forming sequence.

The formation of G-quadruplex DNA structures can prevent the progression of DNA polymerases along DNA. To test if the *BCL6* Cluster III breakpoint region has such ability, the wild-type *BCL6* Cluster III breakpoint region was cloned into pBluescript SK+ (pBC2, Fig. 2*A*, upper panel). A mutant construct was also generated by mutating G-stretches of the *BCL6* Cluster III region (pBC7, Fig. 2*A*, lower panel). Primer extension studies showed the presence of prominent pause sites at the position corresponding to the G4 motif at around 100 nt in wild-type pBC2 when forward primer RT36 was used (Fig. 2*B*, lanes 2–4). Reactions in the presence of increasing KCl concentrations displayed enhanced pause site intensity upon polymerase arrest (Fig. 2*B*, lanes 2–4). While primer extension pausing across the *BCL6* Cluster III region was Potassium dependent, a non-linear response with the strongest pause observed at KCl concentrations (100 mM). This likely reflects an optimal balance between G-quadruplex stabilization and DNA polymerase activity, whereas 150 mM KCl may partially impair polymerase-DNA interactions, reducing extension efficiency and consequently a decrease in the apparent pause signal. Primer extension through the complementary C-rich region using reverse primer RT37 did not show such pause sites (Fig. 2, *B* and *C*, compare lanes 6–8). Besides, in the case of mutant pBC7, no such pause sites were observed with the forward primer (Fig. 2*C*, lanes 2–4).

Assessment of the dependence of structure formation on other cations, such as sodium, lithium, potassium, and calcium, revealed that among the examined cations, potassium had the maximum ability to cause pause sites, followed by sodium (Fig. 2*D*, lanes 3, 9). In contrast, the intensity of the pause site was less in the presence of lithium at both concentrations tested (50 and 100 mM; Fig. 2*D*, lanes 4, 10). In the presence of calcium, the extension reaction was inhibited entirely at both concentrations (Fig. 2*D*, lanes 6, 12). This could be due to inhibition of polymerase activity at CaCl_2_ concentrations tested, rather than enhanced structure formation (29, 30). In contrast, monovalent cations, particularly potassium, induced the strongest pausing followed by sodium, while lithium showed minimal effects, consistent with the established G-quadruplex stabilization hierarchy (K^+^ > Na^+^ ≫ Li^+^). In the presence of an increasing concentration of cationic porphyrin ligand, TMPyP4, [5,10,15,20-Tetrakis-(N-methyl-4-pyridyl) porphine] (31) (Fig. 2*E*), an increase in pause site intensity was observed at the *BCL6* Cluster III (Fig. 2*F*, lanes 2–8). These results suggest that the presence of a G4 DNA on the G-rich strand is potassium-dependent and can be further stabilized by the addition of TmPyP4, leading to a replication pause.

To determine whether additional G4-defective mutants behaved similar to pBC7, we generated an independent mutant construct (pSK2) in which a guanine stretch was mutated in a second G4-forming motif (Fig. 2*G*). Primer extension analysis revealed a marked reduction in replication pausing for the G4 mutant compared with the wild-type *BCL6* Cluster III plasmid (pBC2), as evidenced by loss of the prominent pause site observed in the wild-type construct (Fig. 2*H*). Collectively, these findings demonstrated that disruption of G-quadruplex-forming sequences at *BCL6* Cluster III relieved replication pausing and associated defects, reinforcing the conclusion that these effects were driven by G-quadruplex structure formation.

### Disruption of G-quadruplex structures enhances plasmid propagation in *E. coli*

**Figure 3:**
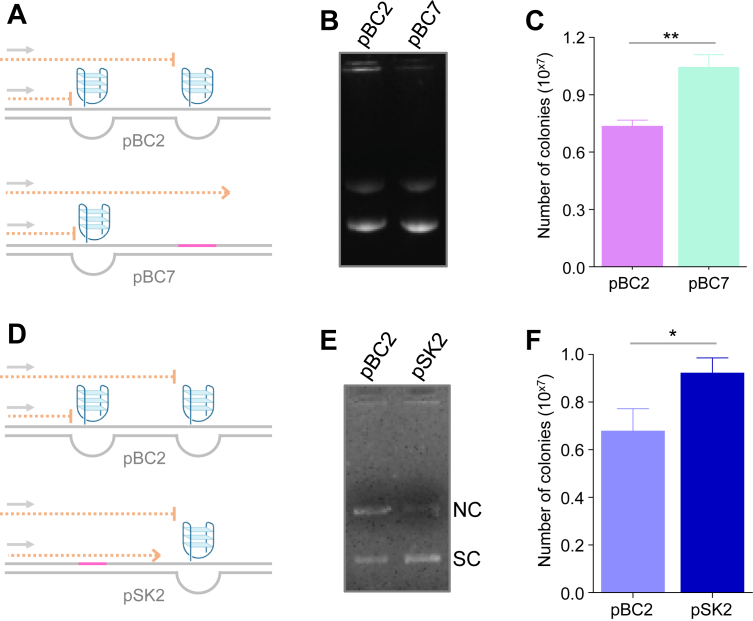
**Effect of G-quadruplex forming sequence mutation on plasmid propagation.***A*, schematic representation of wild-type (pBC2) and G4-mutant (pBC7) plasmids used to assess replication-associated plasmid stability. The wild-type plasmid pBC2 contained the *BCL6* Cluster III G-quadruplex forming region, which induced DNA polymerase pausing, whereas the mutant plasmid pBC7 carried a mutation within a G-rich sequence that disrupted G-quadruplex formation and alleviated polymerase pausing. *B*, agarose gel electrophoresis profile showing the wild-type (pBC2) and mutant (pBC7) plasmids used for bacterial transformation. Comparable band intensities confirmed that equal amounts of plasmid DNA were used. *C*, bar graph depicting quantification of bacterial colonies obtained following transformation of *E. coli* with the wild-type (pBC2) or G4-mutant (pBC7) construct. The G4-mutant plasmid yielded a higher number of colonies than the wild-type construct, indicating enhanced plasmid propagation upon disruption of the G-quadruplex forming sequence. *D*, schematic representation of wild-type (pBC2) and a second G4-mutant (pSK2) plasmids used to assess replication-associated plasmid stability. *E*, agarose gel electrophoresis profile showing the wild-type and mutant (pSK2) plasmids used for bacterial transformation. *F*, bar graph showing quantification of bacterial colonies obtained after transformation of *E. coli* with the wild-type pBC2 or the alternate G4-mutant construct (pSK2). The G4-mutant plasmid (pSK2) consistently produced a higher number of colonies than the wild-type construct.

Primer extension analysis demonstrated that the wild-type *BCL6* Cluster III sequence, harboring G4 motifs, induced DNA polymerase pausing, whereas mutation of the G-quadruplex motif abolished this replication block. To determine whether this pausing impaired plasmid maintenance in bacteria (Fig. 3, *A* and *D*), equal amounts of plasmids carrying the wild-type Cluster III sequence (pBC2) or the G4-mutant sequence (pBC7) were transformed into *E. coli*, and colony numbers were quantified following agarose gel verification of DNA amounts (Fig. 3, *B* and *E*). The G4-mutant plasmid pBC7 consistently produced significantly more bacterial colonies than the wild-type construct, indicating enhanced plasmid propagation upon elimination of the G4-associated replication barrier (Fig. 3*C*).

Consistent with these findings, bacterial transformation assays showed that an additional independent G4-mutant construct (pSK2) also exhibited enhanced plasmid propagation compared to the wild-type pBC2 (Fig. 3*F*). These results demonstrate that replication pausing at *BCL6* Cluster III compromised plasmid propagation in bacteria, while disruption of the G-quadruplex motif restored efficient plasmid maintenance (Fig. 3, *C* and *F*).

### GNG motifs can support intermolecular G-quadruplexes but not intramolecular structure formation

**Figure 4:**
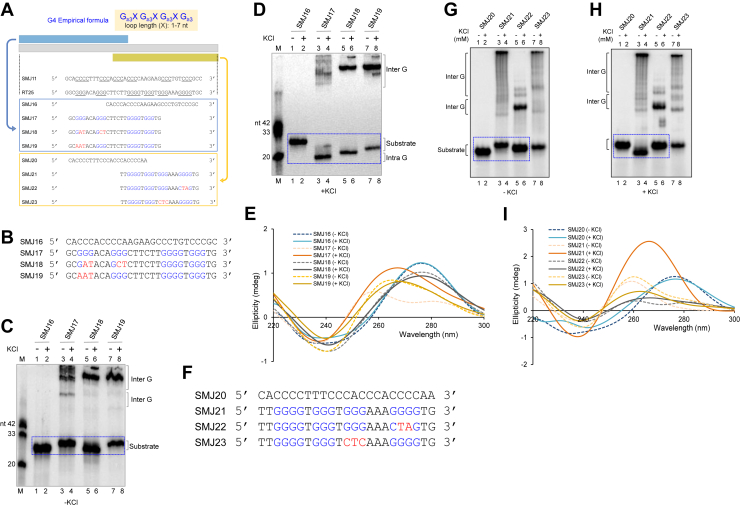
**Empirical G-quadruplex formula and participation of GNG motifs in G4 formation within *BCL6* Cluster III.***A*, classical empirical definition of G-quadruplexes. G denotes guanine residues; X denotes loop of length 1 to 7 nucleotides. In *BCL6* cluster III (RT25), six guanine stretches (*underlined*) are present. Schematic for trimmed sequences (*blue* and *yellow boxed* sections) to accommodate four guanine stretches (*blue*). This allowed evaluation of guanine residues that participate in G4 formation. Mutations (*red*) in the shorter sequences were introduced to determine participation of GNG motifs. *B*, SMJ11-RT25 were shortened from 5′ end such that four stretches of guanines are present in the G-rich (SMJ17). Corresponding C-rich (SMJ16) and mutant sequences (SMJ18, 19) are presented. *C* and *D*, radiolabeled substrates were electrophoresed in 15% native PAGE either in absence (*C*) or presence (*D*) of KCl in gel and running buffer. Lanes 1 to 2 corresponds to C-strand (SMJ16), lanes 3 to 4 correspond to G-strand (SMJ17) and lanes 5 to 8 correspond to the mutants (SMJ18–19). Mobility shifts within the molecules (intramolecular) are marked by a *blue box* while intermolecular structures are labelled. A molecular weight marker consisting of three oligomers (20, 33, and 42 nt) was employed to ensure precise comparison of electrophoretic band migration. *E*, circular dichroism spectra were recorded for the C-strand (SMJ16), G-strand (SMJ17), and mutant substrates (SMJ18–19) in the absence or presence of 100 mM KCl. *F*, sequences SMJ11-RT25 were trimmed from the 3′ end to cover four guanine stretches in the oligomers: G-rich (SMJ21), corresponding C-rich (SMJ20), and mutant sequences (SMJ22, 23). *G* and *H*, electrophoretic mobility shift assays in the absence (*G*) or presence (*H*) of KCl. Lanes 1 to 2, complementary C-strand SMJ20; lanes 3 to 4, wild-type oligomer SMJ21; lanes 5 to 8, mutant oligomers SMJ22 and SMJ23. Mobility shifts corresponding to intramolecular structures are indicated by boxes, while intermolecular structures are labelled. *I*, circular dichroism spectra were recorded for the wild-type G-strand (SMJ21), complementary C-strand (SMJ20), and mutant substrates (SMJ22–23) in the absence or presence of 100 mM KCl. In all panels, CD spectra were acquired at room temperature over the wavelength range 220 to 300 nm using a scan speed of 50 nm/min and are presented as ellipticity (Y-axis) *versus* wavelength (X-axis).

The classical empirical formula of G-quadruplexes accounts for the presence of four guanine stretches with at least three guanine residues each, separated by a loop length of 1 to 7 nucleotides to predict the existence of G4 in the sequence of interest (Fig. 4*A*) (32). In *BCL6* cluster III, six guanine stretches (underlined) are present (Fig. 4*A*). Sequences were shortened to accommodate four guanine stretches (blue and yellow boxed sections), which allowed assessment of the significance of these guanines in G4 formation (Fig. 4*A*). Mutation of these stretches (red) was tested to determine if GNG motifs can participate in G4-structure formation (Fig. 4*B*). Gel mobility shift assay in the absence of KCl showed that the trimmed sequence with four stretches of guanines, SMJ17 (Fig. 4*C*, lanes 3, 4) migrated along with the C-rich control (SMJ16) (Fig. 4C, lanes 1, 2). While mutant SMJ18 also migrated along with SMJ16, the mutant SMJ19 migrated more slowly than SMJ16 (Fig. 4*C*, lanes 5–8). Interestingly, upon the addition of KCl, wild-type SMJ17 (Fig. 4*D*, lanes 3, 4) showed faster migration as compared to C-rich SMJ16 (Fig. 4*D*, lanes 1, 2). SMJ17, SMJ18, and SMJ19 also showed higher molecular weight species, showing the formation of intermolecular G4-structures (Fig. 4*D*, lanes 3–8), though both SMJ18 and SMJ19 showed the formation of new species in a KCl-dependent manner when GNG sequences were forced to use due to the mutation. We also used a molecular weight marker consisting of three oligomers of defined lengths (20, 33, and 42 nt) to enable direct comparison of band migration, which ensured that the observed mobility shifts were attributable to the structural differences rather than size-dependent effects (Fig. 4, *C* and *D*). These observations suggest that the GNG motifs also facilitate intramolecular G4 formation. However, both mutant SMJ18 and SMJ19 did not exhibit any migration shift, suggesting that despite the presence of the GNG motif, an intramolecular G4-structure was not formed. Circular dichroism studies in the absence of Potassium showed that the CD spectrum of SMJ16 has a peak at ∼280 nm and a dip at ∼250 nm. G-rich SMJ17 showed peaks at ∼265 nm and a dip at 240 nm. Mutant SMJ18 had a peak at ∼280 nm and a dip at ∼250 nm, similar to that of B-DNA. However, mutant SMJ19 showed a peak at ∼265 nm, and 285 nm and a dip at 240 nm, as seen for SMJ17 (indicative of a mixed population of G4 species in the absence of KCl) (Figs. 4*E* and S2*A*). Upon addition of KCl, SMJ17, and SMJ19 indicated parallel G4 spectra with positive peaks at ∼265 nm and dips at 240 nm. In contrast, C-rich SMJ16 and mutant SMJ18 retained the single-stranded B-DNA spectral pattern of ∼280 nm peak and ∼250 nm dip (Figs. 4*E* and S2*B*).

For the second set, the trimmed wild-type (SMJ21), corresponding C-rich sequence (SMJ20), and mutant sequences (SMJ22, SMJ23) were analyzed (Fig. 4*F*). Gel mobility shift assays in the absence of Potassium showed no shift in migration for SMJ21 and its mutants compared to the C-rich strand (Fig. 4*G*). In the presence of KCl, the wild type of oligomer, SMJ21 (Fig. 4*H*, lanes 3, 4), migrated faster when compared to C-rich SMJ20, suggesting intramolecular G4 DNA (Fig. 4*H*, lanes 1, 2). Interestingly, such intramolecular G4 DNA formation was absent in the case of both the mutants SMJ22 and SMJ23 (Fig. 4*H*). Higher-molecular-weight bands corresponding to intermolecular G4 species were observed in all the G-rich sequences. They were unaffected by the presence of KCl. Circular dichroism studies in the absence of KCl indicated that C-rich SMJ20 forms a peak at ∼280 nm and a dip at 260 nm. G-rich SMJ21 exhibited a ∼265 nm peak and a 240 nm dip. Mutant SMJ22 had two peaks at 260 nm and ∼280 nm, and a 240 nm dip. Mutant SMJ23 showed a peak at ∼265 nm and a dip at 240 nm (Figs. 4*I* and S2*C*). SMJ21 exhibited a higher positive peak at 265 nm than in the absence of KCl, accompanied by a 240 nm dip, suggesting a parallel G4-structure. Comparable spectra were recorded in the presence of KCl for all sequences (Figs. 4*I* and S2*D*). These results indicate that in the case of mutants, intermolecular G4 forms may have contributed to the CD spectra corresponding to G4 DNA.

Therefore, our results reveal that 6G stretches in the *BCL6* Cluster III can fold into multiple forms of G4 DNA using four of the G stretches at a given time. Additionally, G4 DNA may be formed using GNG stretches instead of the GGG sequence.

### Transcription is affected when *the BCL6* cluster III sequence, which possesses G4 DNA, is positioned upstream of the coding sequence

**Figure 5:**
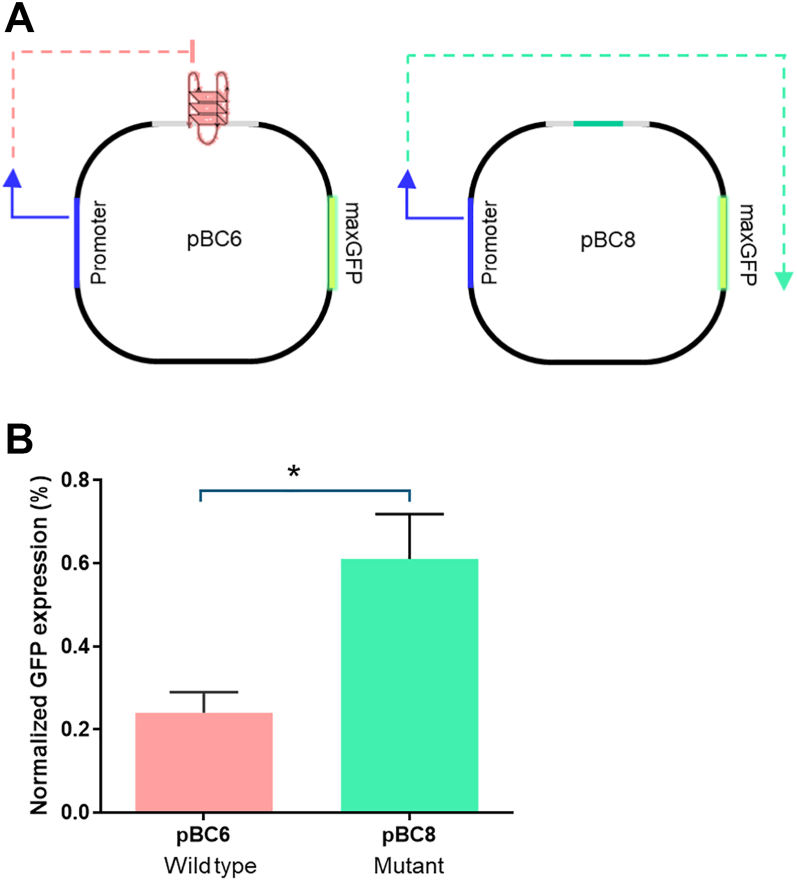
**Potential G-quadruplex formation at *BCL6* Cluster III region within cells.***A*, plasmid constructs, pBC6 and pBC8, were constructed as described in methods. Briefly, wild type or mutant 600 bp fragments containing *BCL6* cluster III were cloned between maxGFP gene and its promoter. Formation of a structure can block transcription through maxGFP gene and thereby reduce or abolish its expression. Absence of structure allows expression of maxGFP gene. *B*, SUDHL8 cells were transfected with both, pBC6 or pBC8, and pCMV-βgal as transfection control. Percentage of GFP expressing cells after transfection was analyzed by flow cytometry, normalized with respective percentage of transfection efficiency, and plotted as bar diagram with error bars (∗*p* < 0.05, ∗∗*p* < 0.01, ∗∗∗*p* < 0.001).

To investigate whether the DNA sequence in cluster III can block gene expression in mammalian cells due to its potential to form a G4 DNA conformation within cells, a reporter assay system based on green fluorescent protein (EGFP) was used. Wild-type and mutant Cluster III regions were cloned upstream of the EGFP gene in the episome pMAXGFP (pBC6 and pBC8, respectively) (Fig. 5*A*) and transfected into SUDHL8 cells. pCMV-β gal construct was co-transfected to determine the transfection efficiency. Upon flow cytometric analysis, a significant decrease in GFP expression was observed in cells transfected with the wild-type construct (pBC6) as compared to the mutant (pBC8) (Fig. 5*B*). Normalized GFP expression of the wild-type construct was approximately two-fold less compared to the mutant (Fig. 5*B*), indicating that the formation of G-quadruplex at *BCL6* Cluster III can affect the transcription within mammalian cells, if unresolved, affecting the protein expression.

### Single-strandedness exists at *the BCL6* cluster III region, complementary to the area that folds into the G4 DNA when present in a plasmid DNA and at the genomic level

**Figure 6:**
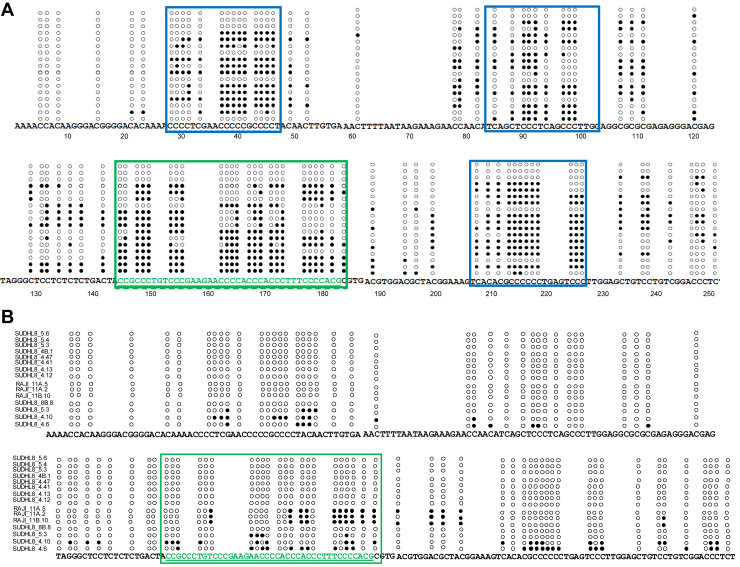
**Sodium bisulfite modification assay of *BCL6* Cluster III region in plasmid and in mammalian genomic DNA.***A*, plasmid pBC2 containing *BCL6* Cluster III was subjected to sodium bisulfite treatment. Each circle represents one cytosine in the *BCL6* Cluster III region. Closed circle denotes cytosine conversion to thymine while open circle denotes unconverted cytosine. Sequence identical to RT25 is indicated and boxed in *green*. Additional regions with stretch conversions have been marked in *blue*. *B*, Genomic DNA was extracted by non-denaturing method and treated with sodium bisulfite as described. Each circle represents one cytosine in the *BCL6* Cluster III breakpoint region. Open circle represents unconverted cytosine while closed circle denotes cytosine conversion to thymine. Region of interest forming the G4-structure is denoted in *green* (*boxed*).

Sodium bisulfite probing of plasmid and cellular genome was conducted to assess the single-strandedness at the *BCL6* Cluster III region (Fig. S3). A sodium bisulfite modification assay was performed in the context of a double-stranded pDNA after cloning the region of interest to generate the pBC2. PCR amplification followed by DNA sequencing revealed that of 20 DNA molecules sequenced, 16 molecules exhibited C to T conversions in stretches (Fig. 6*A*). Some molecules showed conversion in all the cytosine residues complementary to the guanine residues, while others showed sequences complementary to either 5′ or 3′ G-stretches that were involved in the G-quadruplex formation (Fig. 6*A*; sequence indicated in green, boxed). In addition to the C to T conversion observed for the G4 motif of Cluster III, flanking regions also showed the presence of consistent cytosine conversions, suggesting that the sequences upstream or downstream of this region may harbor some non-B DNA motifs, which were undetected using standard non-B DNA prediction tools (Fig. 6*A*, sequences boxed in blue).

Genomic DNA isolated from SUDHL8 (DLBCL cell line) cells was also subjected to the Sodium bisulfite modification assay. Interestingly, single-strandedness was detected in the complementary regions (marked in green—region of interest) corresponding to the break point region, cluster III of the genome (Fig. 6*B*). Some clones did not show these C→T conversions in the cytosine stretches, suggesting that the G-quadruplex structures at *BCL6* Cluster III may not be formed in all cells and may be formed only under specific cellular conditions in the context of the genome.

### Identification of novel DNA structure at the flanking regions of cluster III, which were not predicted by the software

**Figure 7:**
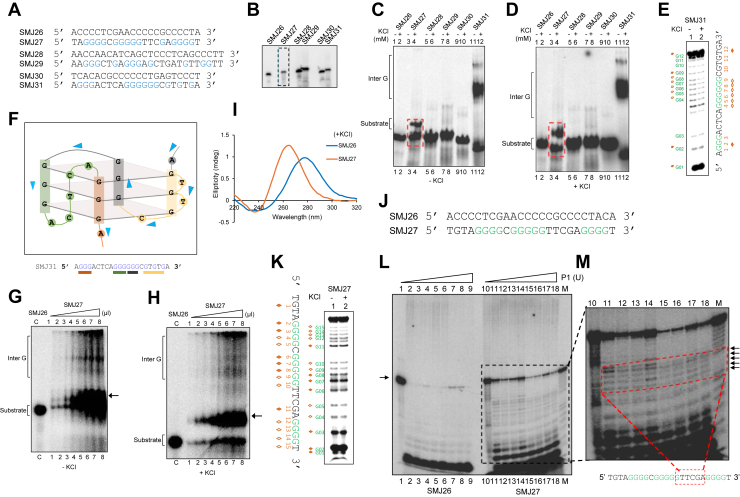
**Native gel electrophoresis and chemical probing of the flanking C-rich and G-rich sequences.***A*, sequences of oligomers used in the assays. Guanine residues are indicated in *blue*. *B*, all the substrates were heat denatured and resolved on 8% denaturing PAGE. *C* and *D*, native gel electrophoresis was performed in absence (*C*) or presence (*D*) of KCl. Lanes 1 to 2, 5 to 6, 9 to 10 represent C-rich strands (SMJ26, SMJ28 and SMJ30 respectively) and lanes 3 to 4, 7 to 8, 11 to 12 represent G-rich strands (SMJ27, SMJ29 and SMJ31). *Red box* represents mobility shift for SMJ27. *E*, DMS probing for SMJ31 showed cleavage at guanine residues (positions marked and numbered) in both absence (lane 1) and presence of KCl (lane 2). Residues G2-G12 (marked with square) showed protection from piperidine cleavage. *F*, representation of a three-plate G4-structure that forms in one region of *BCL6* cluster III. Guanine residues (in *blue*) are denoted in sequence and structure with corresponding bars of identical color. Arrowheads denote strand direction from 5′ to 3′. *G* and *H*, native gel electrophoresis was performed with radiolabeled SMJ27, following incubation at 37 °C in increasing amounts (0.1, 0.2, 0.5, 1.0, 1.5, 2, 2.5, 3.0 μl) and resolved on native polyacrylamide PAGE in absence (*G*) or presence (*H*, 100 mM) of KCl. *Arrow* indicates the shift in mobility in both the panels. *I*, circular dichroism spectra of the sequences SMJ26 and SMJ27 were recorded at room temperature from 220 to 320 nm, at a scan speed of 50 nm/min and plotted as function of wavelength on X-axis, and ellipticity values on Y-axis. *J*, sequences of the oligomers used in the study. Guanines in the G-rich sequence are marked in *green*. *K*, SMJ27 upon incubation in absence and presence of KCl, was treated with DMS at 37 °C for 15 min, followed by piperidine treatment at 90 °C for 30 min. The reaction products were electrophoresed on denaturing PAGE. Cleavage pattern showed protected guanines (denoted by *brown rhombuses*) within the sequence (residues marked in *green*) in absence and presence of KCl. *L* and *M*, SMJ26, C-rich substrate and SMJ27, G-rich substrate was subjected to increasing P1 nuclease concentration (0, 0.05, 0.07, 0.08, 0.09, 0.10, 0.15, 0.18, and 0.20 U) for 30 min at 37 °C and resolved on denaturing PAGE (*L*). SMJ26 (lanes 1–9) showed cleavage by P1 from 0.05 U (lane 2) onwards. Undigested substrate is denoted by arrow. SMJ27 showed P1 cleavage in specific residues (*boxed*). Boxed region (*M*) shows cropped gel image from *panel L*. Lane M is DMS-treated SMJ27, that was electrophoresed with P1-digested SMJ27 (lanes 10–18). Corresponding digested bands in SMJ27 (lanes 10–18) are boxed in red. Arrow-marked residues in DMS-treated SMJ27 (lane M) mark the absent bands for unreacted residues. These residues are plotted in the sequence specified below the gel image (encased in red box).

The additional single-stranded regions observed at the Cluster III breakpoint region, as determined by the Sodium bisulfite modification assay were further analyzed *in silico* for potential non-B DNA structures. However, no structures were predicted despite the presence of guanine residues in these sequences (Fig. 7*A*, guanine marked in blue). Three sets of oligomeric DNAs covering the single-stranded region from Cluster III were designed, synthesized, and electrophoresed on a denaturing PAGE (Fig. 7*B*). Results showed a minor difference in mobility when G-stretch DNA was compared to C-rich DNA. Gel mobility shift assays showed that SMJ27 (Fig. 7*C*, lanes 3, 4; boxed in red) migrated faster as compared to the C-rich control SMJ26 (Fig. 7*C*, lanes 1, 2), suggesting non-B DNA structure formation. SMJ29 (Fig. 7*C*, lanes 7, 8) showed only a minor mobility shift compared to C-rich SMJ28 (Fig. 7*C*, lanes 5, 6). SMJ31 (Fig. 7*C*, lanes 11 and 12) exhibited faster mobility compared to the C-rich SMJ30 (Fig. 7*C*, lanes 9 and 10). The migration pattern of SMJ27 was not entirely indicative of intermolecular G4 form; also, it did not resemble the mobility shift generally observed for the intramolecular G4 forms (Fig. 7*C*, lanes 3,4). Upon addition of KCl, SMJ27 migrated faster (Fig. 7*D*, lanes 3, 4; boxed in red) as compared to C-rich SMJ26 (Fig. 7*D*, lanes 1, 2). SMJ29 (Fig. 7*D*, lanes seven and 8) exhibited a difference in mobility compared to C-rich SMJ28 (Fig. 7*D*, lanes five and 6). SMJ31 (Fig. 7*D*, lanes 11 and 12) exhibited faster mobility compared to C-rich SMJ30 (Fig. 7*D*, lanes 9 and 10). To distinguish size-dependent effects from structural changes, native gels were run with a 20, 33, and 42 nt oligomer ladder as a molecular weight marker. In the absence of KCl, the G-rich strands migrate at positions consistent with their expected lengths, whereas potassium addition results in enhanced mobility relative to the size standards. These potassium dependent shifts reflect structure induced compaction of the G-rich strand, consistent with G-quadruplex formation rather than differences in molecular weight (Fig. S4, *A* and *B*). DMS probing of SMJ31 was conducted to assess the status of Hoogsteen base pairing. Guanine residues (denoted in green) showed protection from DMS reactivity, both in the absence and presence of Potassium (Fig. 7, *E* and *F*). Another sequence, SMJ27, showed a peculiar shift in the gel mobility shift assays. Gel mobility shift assays were performed with SMJ27 in absence and presence of Potassium to delve into the nature of the structure formed (Fig. 7, *G* and *H*). In the absence and presence of KCl, increase in substrate quantity demonstrated a proportional increase in the intensity of the shifted band indicating structure formation. The C-rich control SMJ26 did not exhibit any such change in mobility (Fig. 7, *G* and *H*). CD spectra of SMJ27 showed formation of parallel G4, with a dip at 240 nm and peak at 265 nm; whereas that of C-rich SMJ26 had ∼280 nm positive peak and 260 nm negative dip, typical spectrum of ss B-DNA (Fig. 7*I*). These results established that the non-B DNA formed at this region is a parallel G-quadruplex and has features that make it migrate differently during native gel electrophoresis. DMS assay showed the presence of guanine protection in SMJ27, in the absence and presence of KCl (denoted by brown rhombuses, guanine residues in green) (Fig. 7*K*).

**Figure 8:**
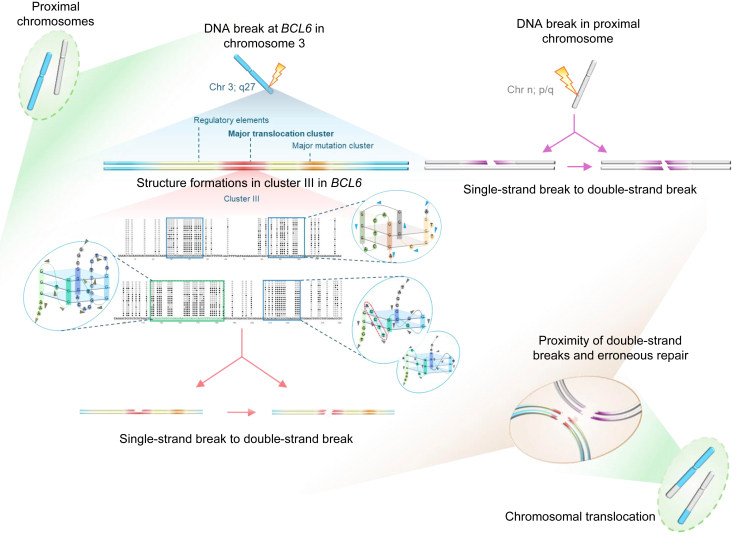
**Proposed model for formation of multiple non-B DNA structures in the *BCL6* cluster III, and their involvement in chromosomal translocations in DLBCL.** In an event of proximal chromosomes within the nuclear territory undergoing DNA breaks (whether single-stranded or converted to double-stranded at later point), the chromosome in *blue* represents chromosome three harboring *BCL6* locus, while the chromosome in *grey* represents partner chromosome, harboring one of the 49 translocation loci. DNA breakage in *BCL6* cluster III region is the result of multiple non-B DNA structures formed during replication or transcription within the sequences, as depicted. Nonresolution of such structures by DNA helicases like Bloom, Werner’, Pif1 can make them ideal substrates for structure-specific endonucleases or deaminase activity of AID activity, rendering fragility. Occurrence of a contemporaneous break in partner chromosomes in nuclear vicinity and subsequent mis repair, through erroneous joining with the DSB in *BCL6* locus, leads to chromosomal translocations, that are one of the genetic hallmarks of DLBCL.

Furthermore, a single-strand specific enzyme, P1 nuclease assay, was performed to assess G4 DNA formation in SMJ27. The C-rich DNA, SMJ26, served as a control in this case. Both SMJ26 and SMJ27 were subjected to increasing concentrations of P1 nuclease and resolved on a denaturing PAGE (Fig. 7*L*). C-rich substrate SMJ26 showed high sensitivity to the P1 nuclease action, as clear from substrate digestion from the lowest P1 concentration onwards (Fig. 7*L*, lanes 2–9). Structure-forming SMJ27 showed a definite pattern of sensitivity. It was not completely digested by P1 even at the highest concentration (Fig. 7*L*, lanes 10–18), indicating the resistance to digestion conferred by the structure. DMS-treated substrate (Fig. 7*M*, lane M) was resolved along with SMJ27 treated with increasing concentration of P1 nuclease (Fig. 7*L* and M, boxed region). This approach helped identify residues in SMJ27, other than guanines, that display sensitivity to P1 nuclease. Interestingly, higher sensitivity was noted in the region that showed no DMS reactivity (Fig. 7*M*, lanes 11–18, M). This result provided an important insight into the structure formed based on the nucleotides in the involved sequence. These residues of SMJ27 (Figs. 8 and S5*A*) could belong to one of the loops of the G-quadruplex structure, if it is of a two-plate nature (Figs. 8 and S5*C*), instead of a G4-form containing three plates (Figs. 8 and S5*B*). The formation of the three-plate G-quadruplex form is also possible in this region; however, the results of the studies so far suggest that the structure is more likely to be of a two-plate nature (Fig. 8).

The findings of the present study revealed that non-B DNA structures, including multiple G-quadruplexes, form at Cluster III, which may contribute to the fragility of breakage-prone regions of the genome, such as the *BCL6* Cluster III present in the major translocation cluster (MTC). Further investigation is required to implicate the role of G-quadruplexes in causing t(3;n) (q27;n) translocation in diffuse large B-cell lymphoma.

## Discussion

The molecular etiology of one of the most common types of non-Hodgkin lymphomas, diffuse large B-cell lymphoma (DLBCL), involves a cascade of transformation and expansion of malignant clones of germinal B cells. The two major contributing molecular alterations in this context are chromosomal translocations (comprising the major translocation cluster, MTC) and somatic hypermutations (comprising the major mutation cluster, MMC) in the *BCL6* locus of DLBCL cases. These molecular alterations are highly relevant to lymphomagenesis. Notably, aberrant somatic hypermutations—present in over 50% of DLBCL cases—arise from dysregulated physiological somatic hypermutation (SHM) in B-cell germinal centers (33, 34). Actively transcribed genes, like proto-oncogenes *PIM1* and *MYC*, show the presence of multiple somatic mutations (35); as expected, though, aberrant SHM is restricted to selected genes like Ig loci and *BCL6* (36, 37), whereas mutations in other off-target genes are commonly fixed with high accuracy (38).

*BCL6* translocations within the major translocation cluster juxtapose the gene with diverse non-Ig or Ig partners, disrupting cellular homeostasis and driving B-cell lymphomagenesis. To elucidate the causes of recurrent fragility at the *BCL6* locus, we analyzed patient breakpoint sequences. Both we and others have identified clustered DNA breakpoints within the 5′ UTR major translocation clusters, delineating three distinct regions: Clusters I, II, and III. Cluster III, which harbors the highest density of patient breakpoints, was the focus of this study.

Genome fragility arises from multiple factors, including DNA sequence/structural features and processes such as replication, transcription, and repair. Among these, non-B DNA structures have been implicated in chromosomal translocations associated with follicular lymphoma, Burkitt lymphoma, T-cell acute lymphoblastic leukemia, and other malignancies (39, 40, 41, 42, 43, 44, 45, 46, 47, 48). Previously, studies from our laboratory revealed that different non-B form of DNA, such as triplexes, cruciform DNA, and G4 DNA, contribute to the fragility of Cluster II of the *BCL6* breakpoint region (15).

In this study, we employed complementary experimental approaches to demonstrate that G-quadruplex (G4) DNA structures contribute to fragility at the *BCL6* Cluster III breakpoint region. Detailed structural analysis revealed that while most reported G4 structures comprise three guanine tetrad layers, Cluster III formed both three-layer and two-layer G4 structures (Fig. 8). The three-layer G4 motif, predicted by non-B DNA databases (49, 50, 51), exhibited parallel topology and potassium dependence. In contrast, the two-layer G4 formed independently of potassium. Two-plate G4 structures are less commonly reported and typically occur in gene promoter regions, where they function as transcriptional repressors, as observed in the c-*MYC* NHE III1 element (52) and the 5′ UTR of the Rb tumor suppressor gene, where they impede DNA polymerase progression (53). Another instance is in *the KRAS* promoter at NHE (54). In the region of interest under study, the formation of a two-plate G4 was detected in one of the breakpoint regions. The observed clustering of patient breakpoint regions in this area suggests that both the G4-structures (two- and three-plate forms) contribute to the fragility of *BCL6* Cluster III. Their differential impact on DNA fragility, if any, could be investigated further by analyzing more DLBCL patient breakpoint sequences in the future.

The role of G-quadruplex DNA has been implicated in both the physiological and pathological functions of the cell. Their critical function in ensuring telomere integrity and regulating molecular processes, such as DNA replication, transcription, and translation, has been well explored (55, 56, 57, 58). Deep sequencing studies map G4 motifs in the human replication origins. Furthermore, the human origin recognition complex (ORC) shows *in vitro* binding with G4-DNA (59), highlighting the role of G4-DNA in initiating DNA replication. However, detrimental consequences due to the unregulated or persistent presence of G4 within the genome, resulting from defective resolutions by DNA helicases such as Bloom, Werner's, and Pif1, are also not uncommon (60, 61, 62, 63). Stable G4-DNA can derail the progression of DNA polymerases and stall replication, inadvertently triggering DNA damage and genomic instability (64, 65, 66, 67). Fragile regions of *BCL2* MBR and *HOX11* that cause chromosomal rearrangements in follicular lymphoma and T-cell leukemia respectively, showed formation of pause sites at respective G4-motifs, indicating that the polymerase was unable to proceed upon encountering these G4-structures formed at the two fragile regions (42, 44). Interestingly, when the human genome was assessed for the presence of G4 motifs *in silico*, a significant correlation was observed between the occurrence of these motifs and the location of fragile genomic regions in almost 50% of genes participating in chromosomal rearrangements of lymphoid cancers (68).

In this study, primer extension assays with *BCL6* Cluster III sequence demonstrated that G-quadruplex formation induced DNA polymerase pausing and compromised plasmid propagation in *E. coli*, while G4 motif mutations restored replication efficiency. These findings highlight the stability of Cluster III G4 structures and their capacity to impede DNA polymerase progression. Thus, G4-mediated replication barriers at oncogenic loci like *BCL6* likely contribute to replication stress and genome instability in cancer. Consistent with this, G4 structures have been associated with increased risk of deleterious genetic events during replication fork stalling or collapse (69). Furthermore, such pauses, when introduced into the genome, could result in single-stranded breaks, which may be converted to DSBs either by replication or by the action of structure-specific endonucleases (70) (Fig. 8). One such enzyme is the RAG complex, found in pre- and pro-B cells. RAGs generally act in a sequence-specific manner, but may recognize and cleave at non-B DNA structures when they are formed adjacent to recombination signal sequences (12, 13, 47, 71). However, considering that the *BCL6* translocation generally occurs in mature B cells at the germinal center, it is less likely that RAG plays a role here. Another prospect is the action of activation-induced cytidine deaminase (AID) on the unpaired cytosines in complementary C-rich regions or DNA junctions, rendering them susceptible to breakage (Fig. 8). Notably, the maximum AID expression is detected in DLBCL among all the TCGA tumors. A progressive increase in its expression is noticed throughout the stages of DLBCL (TCGA, UALCAN Portal (72)). Along these lines, probing *BCL6* Cluster III with sodium bisulfite revealed single-stranded complementary regions, suggesting that these regions may become ideal substrates for AID activity, rendering Cluster III susceptible to fragility and potentially triggering subsequent chromosomal translocations (Fig. 8).

In addition to disrupting DNA replication, the presence of G4 in promoters also impacts transcription, as seen for proto-oncogenes *KRAS* and *c-MYC* (52, 73). Along these lines, the present study employed a GFP reporter assay in mammalian cells to demonstrate the debilitating effect that G4-structure formation in the wild-type sequence has on transcription, which is absent in the mutant sequence.

Taken together, the current findings suggest that the formation of G-quadruplex structures in the genome acts as a hotspot of fragility under specific cellular conditions. Previous studies have also revealed other structural motifs in Cluster II of the *BCL6* major translocation cluster (15). A comprehensive characterization of such structures and their contribution to DNA breakage would help further delineate the molecular mechanisms contributing to fragility in this region and several others in the coming days.

## Experimental procedures

### Enzymes, chemicals, and reagents

Chemical reagents were obtained from Sigma Chemical Co. (and SRL (India). DNA-modifying enzymes were obtained from New England Biolabs. Radioisotope-labeled nucleotides were from BRIT. Culture media were obtained from Sera Laboratory International Limited. FBS and PenStrep were purchased from Gibco BRL.

### Oligomeric DNA

All the oligomeric DNA was gel-purified as described earlier (74, 75). The oligomers used in the *BCL6* Cluster III study are listed in Table S1.

### 5′ end labeling of oligomers

The 5′ end labeling of oligomeric DNA was done using T4 polynucleotide kinase with [γ-^32^P]ATP as described earlier (74, 76). The labelled substrates were purified using Sephadex G25 (Sigma) columns and stored at −20 °C until further use.

### Plasmid construction

The 606 bp region of *the BCL6* cluster III was amplified using primers SP5 and SP6. The amplified product was cloned into pBluescript SK + at *the SalI* site and named pBC2 (pBC-BCL6). The mutated region was generated using the PCR-driven overlap extension method, wherein adenine nucleotides were introduced within two stretches of guanines (76, 77). Primers BC18 and BC19, which are mutated in their respective places, along with SP5 and SP6, were employed for this purpose. This amplified product was cloned into pBluescript SK + at *the SalI* site and named pBC7 (pBC-MBCL6). A second G4 mutant construct was generated by PCR-driven overlap extension, in which adenine substitutions were introduced into one guanine-rich stretch using primers harboring mutation SD3.3 and SD3.4, along with flanking primers SP5 and SP6. The resulting PCR product was cloned into the *SalI* site of pBluescript SK + to generate the plasmid pSK2. The mutant clones were verified by sequencing. The wild-type 606 bp *BCL6* Cluster III region was cloned upstream of the GFP gene in pMAX-GFP at *the KpnI* and *NheI* sites (pBC6). The mutated version was also constructed in the same way and named pBC8. The identity of plasmids was confirmed by DNA sequencing.

### Bioinformatics analysis for screening of G-quadruplex motifs

The BCL6 Cluster III breakpoint region was analyzed for the formation of G-quadruplex structures using the "Non-B DB" database (49, 50, 51). The parameter for the number of repeated guanines was set to either two or three to assess the possibility of forming both two- and three-stranded quadruplex structures.

### Gel mobility shift assay

Radiolabeled oligomers were incubated either in the presence or absence of specified concentrations of salts in Tris–EDTA (TE) buffer (pH 8.0) at 37 °C for 1 h (15, 48). Reaction products were resolved on 12% native polyacrylamide gels with or without 25 mM potassium chloride, as specified, at 150 V, at room temperature. The gels were dried, exposed to a screen, and the signal was detected using a PhosphorImager FLA9000 (Fuji, Japan) as described previously (15, 48). A molecular weight marker consisting of three oligomers (20, 33, and 42 nt) was used to facilitate accurate comparison of electrophoretic band migration.

### DMS protection assay

Radiolabeled oligomers were incubated in TE in the absence or presence of 100 mM potassium chloride or lithium chloride at 37 °C for 1 h. Dimethyl sulfate (DMS) was added to reaction (1/200 dilution) and incubated for 15 min at room temperature, as described previously (44, 50). An equal volume of piperidine (10%) was added to each tube, and the reaction was incubated at 90 °C for 30 min. The reaction was diluted to twice its original volume and dried under vacuum. The resultant pellet was washed thrice with water and dried under vacuum. The reaction products were resolved on an 18% denaturing polyacrylamide gel, which was further dried and visualized as described above.

### Circular dichroism (CD)

CD studies were performed as described before. *BCL6* (40, 78) cluster III wild-type and mutant oligomers were incubated either in the presence or absence of potassium chloride (100 mM) in TE at 37 °C for 1 h. Circular dichroism spectra were recorded at room temperature at a wavelength range of 220 to 300 nm (10 cycles) for every sample, using a spectropolarimeter (JASCO J-810) at a scan speed of 50 nm/min. A separate spectrum was measured for buffer alone (15 cycles) and was subtracted from all the experimental spectra. For the abolition of structure, spectra were recorded for the samples incubated with TE and specified salt at 95 °C. The ellipticity was calculated using Spectra Manager and plotted as a function of wavelength.

### Primer extension assay

The presence of replication blocks due to structure formation at the *BCL6* breakpoint region was studied in plasmids pBC2, pBC7 and pSK2 using primer extension (45, 79). Reactions were carried out by mixing 100 ng of DNA sample in (1X) Thermo polymerase buffer [10 mM KCl, 10 mM (NH4)_2_SO_4_, 20 mM Tris-HCl [pH 8.8], 4 mM MgSO_4_ and 0.1% Triton X-100], 4 mM MgSO_4_, 200 μM dNTPs, 0.5 μM end labelled oligomers and 1 U Vent (exo-) polymerase. Linear amplification primer extensions were carried out in a PCR machine (3 × 5 cycles) under the following conditions: 95 °C for 3 min (1 cycle), 94 °C for 45 s, 58 to 64 °C for 45 s (as specified) and 72 °C for 45 s and final extension for 3 min. The annealing temperatures of the primers used were 64 °C for RT36 and 60 °C for RT37. Reactions were terminated by adding a dye containing formamide, and the products were resolved on an 8% denaturing polyacrylamide gel. The gel was dried, and signals were detected using a phosphorImager.

### G-quadruplex dependent effect on plasmid propagation

Equal amounts of wild-type and mutant plasmids (pBC2, pBC7, and pSK2) were verified by electrophoresis on a 0.8% agarose gel. The plasmids were diluted 1000-fold, and ∼10 pg of each was transformed into *E. coli* DH5α competent cells by electroporation using standard procedures. Following transformation, cells were recovered in LB medium for 45 min at 37 °C, after which a 1:5 dilution was plated on LB agar containing ampicillin. Plates were incubated overnight at 37 °C, and colonies were counted after 14 h. Transformation efficiencies were calculated by normalizing colony counts to the corresponding dilution factors and were used for comparative analysis. Bacterial colony numbers obtained after transformation of *E. coli* with equal amounts of the wild-type *BCL6* Cluster III plasmid (pBC2) or the G4-mutant plasmids (pBC7 and pSK2) were quantified and plotted as a bar graph using GraphPad Prism software.

### P1 nuclease assay

P1 nuclease assay was conducted as described before, with slight modifications (77, 80). The oligomers were incubated with increasing concentrations of P1 nuclease as specified. P1 reactions were performed in P1 nuclease buffer (50 mM NaCl, 10 mM Tris-HCl [pH 7.9], 10 mM MgCl_2_, 1 mM DTT) at 37 °C for 30 min. The reaction was stopped by heating at 65 °C for 20 min after the addition of EDTA (10 mM) and formamide dye (1×). The oligomeric products were then loaded onto 15% denaturing polyacrylamide gels, whereas the plasmid reaction products were resolved on an 8% denaturing polyacrylamide gel, electrophoresed, dried, exposed, and scanned using a PhosphorImager FLA9000.

### Sodium bisulfite modification assay

Sodium bisulfite assay was performed as described (41, 47). Briefly, chromosomal DNA was isolated from SUDHL8 and Raji cell lines using a non-denaturing method. Approximately 5 μg of DNA was incubated in 12.5 μl of 20 mM hydroquinone and 457.5 μl of 2.5 M sodium bisulfite [pH 5.2] for 16 to 20 h at 37 °C and purified using the Wizard DNA Clean-Up Kit (Promega). Bisulfite-modified DNA was desulfonated with 0.3 M NaOH at 37 °C for 15 min, ethanol precipitated and resuspended in 30 μl TE buffer. Similarly, plasmid DNA containing the *BCL6* cluster III was also processed similarly. The *BCL6* cluster III breakpoint region from both the treated DNA was PCR amplified, resolved on agarose gel, purified, TA cloned, and sequenced, and plotted conveniently to denote the converted and unconverted cytosines within the sequence.

### Assay to detect altered DNA structure within cells

SUDHL8 cells were transfected with pBC6 or pBC8 by electroporation (400V, 850 μF) along with pCMV-β gal (a kind gift from Prof. K.N. Balaji, IISc, Bangalore) (81, 82). The number of GFP-positive cells in each case was analyzed by flow cytometry, and the transfection efficiency was calculated by counting X-gal-stained cells. The percentage of GFP expression was normalized with β-gal expressing cells for both wild-type and mutant plasmids. Data were analyzed using GraphPad Prism software and plotted as a bar diagram with the standard error of the mean.

### X-gal staining

The transfected cells were harvested and washed with PBS. Further, they were incubated in PBS with (0.2%) glutaraldehyde for 15 min at 37 °C. After washing twice with PBS containing 2 mM MgCl_2_ and (0.02%) NP40, the cells were incubated with PBS containing 5 mM potassium ferrocyanide, 5 mM potassium ferricyanide, 2 mM MgCl_2_, and 1 mg/ml X-gal, overnight at 37 °C. X-gal-stained cells were counted, and the transfection efficiency was calculated.

### Statistical analysis

Statistical analysis was performed using a two-tailed Student’s *t* test in GraphPad Prism (version 5.1; GraphPad Software) to evaluate the significance of differences between groups. A *p*-value of <0.05 was considered statistically significant. All data are presented as mean ± standard error of the mean (SEM).

## Data availability

All data supporting the findings of this study are available in the primary and supplementary figures and tables.

## Supporting information

This article contains supporting information (83).

## Conflict of interest

The authors declare that they do not have any conflicts of interest with the content of this article.

## Supporting information

ClusterIII_Manuscript_Supplemental_text

## References

[1] Akkaya B., Salim O., Akkaya H., Ozcan M., Yucel O.K., Erdem R. (2016). C-MYC and BCL2 translocation frequency in diffuse large B-cell lymphomas: a study of 97 patients. Indian J. Pathol. Microbiol..

[2] Berhan A., Almaw A., Damtie S., Solomon Y. (2025). Diffuse large B cell lymphoma (DLBCL): epidemiology, pathophysiology, risk stratification, advancement in diagnostic approaches and prospects: narrative review. Discover Oncol..

[3] Salam D., Thit E.E., Teoh S.H., Tan S.Y., Peh S.C., Cheah S.C. (2020). C-MYC, BCL2 and BCL6 translocation in B-cell non-hodgkin lymphoma cases. J. Cancer.

[4] Ye B.H., Cattoretti G., Shen Q., Zhang J., Hawe N., de Waard R. (1997). The BCL-6 proto-oncogene controls germinal-centre formation and Th2-type inflammation. Nat. Genet..

[5] Dent A.L., Shaffer A.L., Yu X., Allman D., Staudt L.M. (1997). Control of inflammation, cytokine expression, and germinal center formation by BCL-6. Science.

[6] Cattoretti G., Chang C.C., Cechova K., Zhang J., Ye B.H., Falini B. (1995). BCL-6 protein is expressed in germinal-center B cells. Blood.

[7] Onizuka T., Moriyama M., Yamochi T., Kuroda T., Kazama A., Kanazawa N. (1995). BCL-6 gene product, a 92- to 98-kD nuclear phosphoprotein, is highly expressed in germinal center B cells and their neoplastic counterparts. Blood.

[8] Klein U., Dalla-Favera R. (2008). Germinal centres: role in B-cell physiology and malignancy. Nat. Rev. Immunol..

[9] Hatzi K., Melnick A. (2014). Breaking bad in the germinal center: how deregulation of BCL6 contributes to lymphomagenesis. Trends Mol. Med..

[10] Cardenas M.G., Oswald E., Yu W., Xue F., MacKerell A.D., Melnick A.M. (2017). The expanding role of the BCL6 oncoprotein as a cancer therapeutic target. Clin. Cancer Res..

[11] Kramer M.H., Hermans J., Wijburg E., Philippo K., Geelen E., van Krieken J.H. (1998). Clinical relevance of BCL2, BCL6, and MYC rearrangements in diffuse large B-cell lymphoma. Blood.

[12] Raghavan S.C., Lieber M.R. (2004). Chromosomal translocations and non-B DNA structures in the human genome. Cell Cycle.

[13] Raghavan S.C., Lieber M.R. (2006). DNA structures at chromosomal translocation sites. BioEssays.

[14] Ye B.H., Lista F., Lo Coco F., Knowles D.M., Offit K., Chaganti R.S. (1993). Alterations of a zinc finger-encoding gene, BCL-6, in diffuse large-cell lymphoma. Science.

[15] Gopalakrishnan V., Roy U., Srivastava S., Kariya K.M., Sharma S., Javedakar S.M. (2024). Delineating the mechanism of fragility at BCL6 breakpoint region associated with translocations in diffuse large B cell lymphoma. Cell Mol. Life Sci..

[16] Jardin F., Ruminy P., Bastard C., Tilly H. (2007). The BCL6 proto-oncogene: a leading role during germinal center development and lymphomagenesis. Pathol. Biol. (Paris).

[17] Chaganti S.R., Chen W., Parsa N., Offit K., Louie D.C., Dalla-Favera R. (1998). Involvement of BCL6 in chromosomal aberrations affecting band 3q27 in B-cell Non-Hodgkin lymphoma. Genes Chromosomes Cancer.

[18] Chaganti S.R., Rao P.H., Chen W., Dyomin V., Jhanwar S.C., Parsa N.Z. (1998). Deregulation of BCL6 in Non-Hodgkin lymphoma by insertion of IGH sequences in complex translocations involving band 3q27. Genes Chromosomes Cancer.

[19] Fenton J.A., Schuuring E., Barrans S.L., Banham A.H., Rollinson S.J., Morgan G.J. (2006). t(3;14)(p14;q32) results in aberrant expression of FOXP1 in a case of diffuse large B-cell lymphoma. Genes Chromosomes Cancer.

[20] Galiegue-Zouitina S., Quief S., Hildebrand M.P., Denis C., Detourmignies L., Lai J.L. (1999). Nonrandom fusion of L-plastin(LCP1) and LAZ3(BCL6) genes by t(3;13)(q27;q14) chromosome translocation in two cases of B-cell Non-Hodgkin lymphoma. Genes Chromosomes Cancer.

[21] Hosokawa Y., Maeda Y., Ichinohasama R., Miura I., Taniwaki M., Seto M. (2000). The Ikaros gene, a central regulator of lymphoid differentiation, fuses to the BCL6 gene as a result of t(3;7)(q27;p12) translocation in a patient with diffuse large B-cell lymphoma. Blood.

[22] Kurata M., Maesako Y., Ueda C., Nishikori M., Akasaka T., Uchiyama T. (2002). Characterization of t(3;6)(q27;p21) breakpoints in B-cell non-hodgkin's lymphoma and construction of the histone H4/BCL6 fusion gene, leading to altered expression of Bcl-6. Cancer Res..

[23] Nakamura Y., Takahashi N., Kakegawa E., Yoshida K., Ito Y., Kayano H. (2008). The GAS5 (growth arrest-specific transcript 5) gene fuses to BCL6 as a result of t(1;3)(q25;q27) in a patient with B-cell lymphoma. Cancer Genet. Cytogenet..

[24] Ueda C., Akasaka T., Kurata M., Maesako Y., Nishikori M., Ichinohasama R. (2002). The gene for interleukin-21 receptor is the partner of BCL6 in t(3;16)(q27;p11), which is recurrently observed in diffuse large B-cell lymphoma. Oncogene.

[25] Ueda C., Uchiyama T., Ohno H. (2002). Immunoglobulin (Ig)/BCL6 *versus* non-Ig/BCL6 gene fusion in diffuse large B-cell lymphoma corresponds to a high- *versus* low-level expression of BCL6 mRNA. Blood.

[26] Xu W.S., Liang R.H., Srivastava G. (2000). Identification and characterization of BCL6 translocation partner genes in primary gastric high-grade B-cell lymphoma: heat shock protein 89 alpha is a novel fusion partner gene of BCL6. Genes Chromosomes Cancer.

[27] Yonetani N., Akasaka T., Akasaka H., Ohno H., Okuma M., Miura I. (1998). Molecular features of a new human lymphoma cell line carrying both BCL2 and BCL6 gene rearrangements. Oncogene.

[28] Yoshida S., Kaneita Y., Aoki Y., Seto M., Mori S., Moriyama M. (1999). Identification of heterologous translocation partner genes fused to the BCL6 gene in diffuse large B-cell lymphomas: 5'-RACE and LA - PCR analyses of biopsy samples. Oncogene.

[29] Freudenthal B.D., Beard W.A., Shock D.D., Wilson S.H. (2013). Observing a DNA polymerase choose right from wrong. Cell.

[30] Swan M.K., Johnson R.E., Prakash L., Prakash S., Aggarwal A.K. (2009). Structural basis of high-fidelity DNA synthesis by yeast DNA polymerase delta. Nat. Struct. Mol. Biol..

[31] Phan A.T., Kuryavyi V., Gaw H.Y., Patel D.J. (2005). Small-molecule interaction with a five-guanine-tract G-quadruplex structure from the human MYC promoter. Nat. Chem. Biol..

[32] Burge S., Parkinson G.N., Hazel P., Todd A.K., Neidle S. (2006). Quadruplex DNA: sequence, topology and structure. Nucleic Acids Res..

[33] Iqbal J., Greiner T.C., Patel K., Dave B.J., Smith L., Ji J. (2007). Distinctive patterns of BCL6 molecular alterations and their functional consequences in different subgroups of diffuse large B-cell lymphoma. Leukemia.

[34] Schneider C., Pasqualucci L., Dalla-Favera R. (2011). Molecular pathogenesis of diffuse large B-cell lymphoma. Semin. Diagn. Pathol..

[35] Pasqualucci L., Neumeister P., Goossens T., Nanjangud G., Chaganti R.S., Kuppers R. (2001). Hypermutation of multiple proto-oncogenes in B-cell diffuse large-cell lymphomas. Nature.

[36] Pasqualucci L., Migliazza A., Fracchiolla N., William C., Neri A., Baldini L. (1998). BCL-6 mutations in normal germinal center B cells: evidence of somatic hypermutation acting outside Ig loci. Proc. Natl. Acad. Sci. U. S. A..

[37] Shen H.M., Peters A., Baron B., Zhu X., Storb U. (1998). Mutation of BCL-6 gene in normal B cells by the process of somatic hypermutation of Ig genes. Science.

[38] Liu M., Duke J.L., Richter D.J., Vinuesa C.G., Goodnow C.C., Kleinstein S.H. (2008). Two levels of protection for the B cell genome during somatic hypermutation. Nature.

[39] Bacolla A., Jaworski A., Connors T.D., Wells R.D. (2001). Pkd1 unusual DNA conformations are recognized by nucleotide excision repair. J. Biol. Chem..

[40] Javadekar S.M., Yadav R., Raghavan S.C. (2018). DNA structural basis for fragility at peak III of BCL2 major breakpoint region associated with t(14;18) translocation. Biochim. Biophys. Acta Gen. Subj..

[41] Kumari N., Das K., Sharma S., Dahal S., Desai S.S., Roy U. (2023). Evaluation of potential role of R-loop and G-quadruplex DNA in the fragility of c-MYC during chromosomal translocation associated with Burkitt's lymphoma. J. Biol. Chem..

[42] Nambiar M., Goldsmith G., Moorthy B.T., Lieber M.R., Joshi M.V., Choudhary B. (2011). Formation of a G-quadruplex at the BCL2 major breakpoint region of the t(14;18) translocation in follicular lymphoma. Nucleic Acids Res..

[43] Nambiar M., Raghavan S.C. (2012). Mechanism of fragility at BCL2 gene minor breakpoint cluster region during t(14;18) chromosomal translocation. J. Biol. Chem..

[44] Nambiar M., Srivastava M., Gopalakrishnan V., Sankaran S.K., Raghavan S.C. (2013). G-quadruplex structures formed at the HOX11 breakpoint region contribute to its fragility during t(10;14) translocation in T-cell leukemia. Mol. Cell. Biol..

[45] Paranjape A.M., Desai S.S., Nishana M., Roy U., Nilavar N.M., Mondal A. (2022). Nonamer dependent RAG cleavage at CpGs can explain mechanism of chromosomal translocations associated to lymphoid cancers. PLoS Genet..

[46] Raghavan S.C., Houston S., Hegde B.G., Langen R., Haworth I.S., Lieber M.R. (2004). Stability and strand asymmetry in the non-B DNA structure at the bcl-2 major breakpoint region. J. Biol. Chem..

[47] Raghavan S.C., Swanson P.C., Wu X., Hsieh C.L., Lieber M.R. (2004). A non-B-DNA structure at the Bcl-2 major breakpoint region is cleaved by the RAG complex. Nature.

[48] Sharma S., Thomas E., Dahal S., Das S., Kothari S., Roy U. (2025). Formation of multiple G-quadruplexes contributes toward BCR fragility associated with chronic myelogenous leukemia. Nucleic Acids Res..

[49] Cer R.Z., Donohue D.E., Mudunuri U.S., Temiz N.A., Loss M.A., Starner N.J. (2013). Non-B DB v2.0: a database of predicted non-B DNA-forming motifs and its associated tools. Nucleic Acids Res..

[50] Dahal S., Siddiqua H., Katapadi V.K., Iyer D., Raghavan S.C. (2022). Characterization of G4 DNA formation in mitochondrial DNA and their potential role in mitochondrial genome instability. FEBS J..

[51] Zhao Z., Wang J., Yu H., Wang X. (2024). Guide for phenotype-specific profiling of DNA G-quadruplex-regulated genes. STAR Protoc..

[52] Siddiqui-Jain A., Grand C.L., Bearss D.J., Hurley L.H. (2002). Direct evidence for a G-quadruplex in a promoter region and its targeting with a small molecule to repress c-MYC transcription. Proc. Natl. Acad. Sci. U. S. A..

[53] Xu Y., Sugiyama H. (2006). Formation of the G-quadruplex and i-motif structures in retinoblastoma susceptibility genes (Rb). Nucleic Acids Res..

[54] Cogoi S., Paramasivam M., Filichev V., Geci I., Pedersen E.B., Xodo L.E. (2009). Identification of a new G-quadruplex motif in the KRAS promoter and design of pyrene-modified G4-decoys with antiproliferative activity in pancreatic cancer cells. J. Med. Chem..

[55] Bochman M.L., Paeschke K., Zakian V.A. (2012). DNA secondary structures: stability and function of G-quadruplex structures. Nat. Rev. Genet..

[56] Paeschke K., Simonsson T., Postberg J., Rhodes D., Lipps H.J. (2005). Telomere end-binding proteins control the formation of G-quadruplex DNA structures *in vivo*. Nat. Struct. Mol. Biol..

[57] Rhodes D., Lipps H.J. (2015). G-quadruplexes and their regulatory roles in biology. Nucleic Acids Res..

[58] Sato K., Knipscheer P. (2023). G-quadruplex resolution: from molecular mechanisms to physiological relevance. DNA Repair.

[59] Hoshina S., Yura K., Teranishi H., Kiyasu N., Tominaga A., Kadoma H. (2013). Human origin recognition complex binds preferentially to G-quadruplex-preferable RNA and single-stranded DNA. J. Biol. Chem..

[60] Chatterjee S., Zagelbaum J., Savitsky P., Sturzenegger A., Huttner D., Janscak P. (2014). Mechanistic insight into the interaction of BLM helicase with intra-strand G-quadruplex structures. Nat. Commun..

[61] Hegedus L., Toth A., Harami G.M., Palinkas J., Karatayeva N., Sajben-Nagy E. (2024). Werner helicase interacting protein 1 contributes to G-quadruplex processing in human cells. Sci. Rep..

[62] Huber M.D., Lee D.C., Maizels N. (2002). G4 DNA unwinding by BLM and Sgs1p: substrate specificity and substrate-specific inhibition. Nucleic Acids Res..

[63] Wu W.Q., Hou X.M., Li M., Dou S.X., Xi X.G. (2015). BLM unfolds G-quadruplexes in different structural environments through different mechanisms. Nucleic Acids Res..

[64] Lemmens B., van Schendel R., Tijsterman M. (2015). Mutagenic consequences of a single G-quadruplex demonstrate mitotic inheritance of DNA replication fork barriers. Nat. Commun..

[65] Lopes J., Piazza A., Bermejo R., Kriegsman B., Colosio A., Teulade-Fichou M.P. (2011). G-quadruplex-induced instability during leading-strand replication. EMBO J..

[66] Rider S.D., Gadgil R.Y., Hitch D.C., Damewood F.J.T., Zavada N., Shanahan M. (2022). Stable G-quadruplex DNA structures promote replication-dependent genome instability. J. Biol. Chem..

[67] Sato K., Martin-Pintado N., Post H., Altelaar M., Knipscheer P. (2021). Multistep mechanism of G-quadruplex resolution during DNA replication. Sci. Adv..

[68] Katapadi V.K., Nambiar M., Raghavan S.C. (2012). Potential G-quadruplex formation at breakpoint regions of chromosomal translocations in cancer may explain their fragility. Genomics.

[69] Paeschke K., Capra J.A., Zakian V.A. (2011). DNA replication through G-quadruplex motifs is promoted by the Saccharomyces cerevisiae Pif1 DNA helicase. Cell.

[70] Lambert S., Carr A.M. (2013). Impediments to replication fork movement: stabilisation, reactivation and genome instability. Chromosoma.

[71] Nishana M., Raghavan S.C. (2012). A non-B DNA can replace heptamer of V(D)J recombination when present along with a nonamer: implications in chromosomal translocations and cancer. Biochem. J..

[72] Chandrashekar D.S., Bashel B., Balasubramanya S.A.H., Creighton C.J., Ponce-Rodriguez I., Chakravarthi B. (2017). UALCAN: a portal for facilitating tumor subgroup gene expression and survival analyses. Neoplasia.

[73] Cogoi S., Xodo L.E. (2006). G-quadruplex formation within the promoter of the KRAS proto-oncogene and its effect on transcription. Nucleic Acids Res..

[74] Chiruvella K.K., Sebastian R., Sharma S., Karande A.A., Choudhary B., Raghavan S.C. (2012). Time-dependent predominance of nonhomologous DNA end-joining pathways during embryonic development in mice. J. Mol. Biol..

[75] Sebastian R., Raghavan S.C. (2016). Induction of DNA damage and erroneous repair can explain genomic instability caused by endosulfan. Carcinogenesis.

[76] Dahal S., Siddiqua H., Sharma S., Babu R.K., Rathore D., Sharma S. (2022). Unleashing a novel function of endonuclease G in mitochondrial genome instability. eLife.

[77] Raghavan S.C., Swanson P.C., Ma Y., Lieber M.R. (2005). Double-strand break formation by the RAG complex at the bcl-2 major breakpoint region and at other non-B DNA structures *in vitro*. Mol. Cell. Biol..

[78] Kumari N., Vartak S.V., Dahal S., Kumari S., Desai S.S., Gopalakrishnan V. (2019). G-quadruplex structures contribute to differential radiosensitivity of the human genome. iScience.

[79] Kumari R., Nambiar M., Shanbagh S., Raghavan S.C. (2015). Detection of G-quadruplex DNA using primer extension as a tool. PLoS One.

[80] Nambiar M., Raghavan S.C. (2011). How does DNA break during chromosomal translocations?. Nucleic Acids Res..

[81] Kumari R., Roy U., Desai S., Nilavar N.M., Van Nieuwenhuijze A., Paranjape A. (2021). MicroRNA miR-29c regulates RAG1 expression and modulates V(D)J recombination during B cell development. Cell Rep..

[82] Roy U., Desai S.S., Kumari S., Bushra T., Choudhary B., Raghavan S.C. (2024). Understanding the role of miR-29a in the regulation of RAG1, a gene associated with the development of the immune system. J. Immunol..

[83] Raghavan S.C., Tsai A., Hsieh C.L., Lieber M.R. (2006). Analysis of non-B DNA structure at chromosomal sites in the mammalian genome. Methods Enzymol.

