# Supplementary material for: Canonical and noncanonical forms of G4 DNA at cluster III of the BCL6 breakpoint region could lead to chromosomal translocation in DLBCL
Source: J Biol Chem. 2026 Mar 9;302(5):111347. doi: 10.1016/j.jbc.2026.111347 (PMC13084406; doi:10.1016/j.jbc.2026.111347)
Supplement: ClusterIII_Manuscript_Supplemental_text [file mmc1.doc]

**Supplementary MATERIALS**

**Supplementary figure legends**

**Table S1. List of oligomers used in the present study.**

**Figure S1. *BCL6* gene organization and distribution of DLBCL patient breakpoints.** **A.** *BCL6* gene is 26 kb long, located in chromosome 3, and contains 10 exons with start codon in exon 3. Blue boxes are coding regions, and orange boxes are non-coding regions. Major translocation cluster (MTC) (3.3 kb) harbors the breakpoints reported in patient samples. Major mutation cluster (MMC) (800 bp) contains somatic hypermutation points and an overall high mutation rate. Regulatory elements have 5' non-coding region with silencer activity (exon and intronic sequences) along with p53-response element. **B.** Region within major translocation cluster was analysed for breakpoints in several reported patient samples. Arrows indicate break points within sequence. For convenience of this study, three distinct groups of breakpoints were classified as Cluster I, Cluster II, and Cluster III (Gopalakrishnan et al., 2024). **C.** Bioinformatics analysis by non-B DNA database of *BCL6* breakpoint region revealed presence of inverted repeats, direct repeats, Z-DNA motifs, and potential G quadruplex structure forming sequences in the breakpoint region. Relevant G quadruplex motif in Cluster III sequence is indicated in red box.

**Figure S2. Circular dichroism analysis of C- rich, G-rich and mutant substrates in the absence and presence of KCl. A, B.** Circular dichroism studies in absence (A) or presence (B) of 100 mM KCl for C-strand (SMJ16), G-strand (SMJ17) and the mutant substrates (SMJ18-19). C, D. Circular dichroism studies in absence (C) or presence (D) of 100 mM KCl for wild type G-strand (SMJ21), complementary C-strand (SMJ20) and mutant substrates (SMJ22-23). In all panels, all spectra were recorded at room temperature from 220 to 300 nm, at a scan speed of 50 nm/min and plotted as function of wavelength on X-axis, and ellipticity on Y-axis.

**Figure S3. Sodium bisulfite probing of DNA. A.** Bisulfite moiety of sodium bisulfite sulphonates the C5‐C6 double bond of cytosine by a reversible reaction forming cytosine sulfonate, in acidic pH 5.2. Cytosine sulfonate is hydrolytically deaminated to irreversibly form uracil sulfonate, which upon desulfonation forms uracil, at pH 8.0. Upon PCR amplification and DNA sequencing, this C to U conversion is read as C to T. (Adapted from Raghavan et al., 2006). **B-D.** Variation in single-strandedness of DNA, due to structure formation, transient nature due to DNA breathing, or absence of unpaired bases, due to complete base-pairing, leads to variation in the conversion rates across the molecules sequenced. This method provides single-molecule resolution of the degree of single-strandedness within region of interest.

**Figure S4. Native gel analysis of C- and G-rich strands in presence of KCl. A-B.** Native gel electrophoresis was performed in the absence (A) or presence (B) of KCl. Lanes 1 and 2 correspond to the C-rich strand SMJ26, while lanes 3-4 correspond to the G-rich strands SMJ27. The mobility shift observed for SMJ27 has been boxed. For precise comparison of band migration, a molecular weight marker containing 20, 33, and 42 nt oligonucleotides is also presented.

**Figure S5. Proposed two-plate G-quadruplex structure at BCL6 cluster III.** **A.** Sequences used in the study; guanines marked in green. **B.** A classical three-plate, parallel-oriented G4-structure proposed to form in one flanking region of BCL6 cluster III upon sequence analysis. **C.** A variant of two-plate G4-structure that accommodates the ‘TTCGA’ loop (boxed in red). Guanine residues (enlarged) are denoted in sequences and structures with corresponding bars of identical colors. Arrowheads denote strand polarity from 5’ to 3’.
