# Supplementary material for: Canonical and noncanonical forms of G4 DNA at cluster III of the BCL6 breakpoint region could lead to chromosomal translocation in DLBCL
Source: J Biol Chem. 2026 Mar 9;302(5):111347. doi: 10.1016/j.jbc.2026.111347 (PMC13084406; doi:10.1016/j.jbc.2026.111347)

Table S1

BC13	5′ACCCCTTTCCCACCCACCC 3′
BC14	5′GGGTGGGTGGGAAAGGGGT 3′
BC15	5′AAATGGGTGGGAAAGGGGT 3′
BC16	5′AAATAAATGGGAAAGGGGT 3′
BC17	5′GGGTGGGTGGGAAAAAAAT 3′
SMJ11	5′GCACCCCTTTCCCACCCACCCCAAGAAGCCCTGTCCCGCC 3′
RT25	5′GGCGGGACAGGGCTTCTTGGGGTGGGTGGGAAAGGGGTGC 3′
RT26	5′GGCGATACAGCTCTTCTTGGGGTGGGTGGGAAAGGGGTGC 3′
RT27	5′GGCGGGACAGGGCTTCTTGGGGTGGGTGTAAAAGTAGTGC 3′
RT28	5′GGCGATACAGCTCTTCTTGGGGTGGGTGTAAAAGTAGTGC 3′
RT29	5′GGCGGGACAGGGCTTCTTCGAGTGATTGGGAAAGGGGTGC 3′
RT36	5′ GCCAGAAATCCCGCC 3′
RT37	5′TTGGTGTTCCCTGCC 3′
SP5	5′GTCAGTCGACGAGCCGAACCGAGATTTG 3′
SP6	5′GCATGTCGACCGCCCAAGGTAAGGACCTC 3′
SMJ16	5′CACCCACCCCAAGAAGCCCTGTCCCGC 3′
SMJ17	5′GCGGGACAGGGCTTCTTGGGGTGGGTG 3′
SMJ18	5′GCGATACAGCTCTTCTTGGGGTGGGTG 3′
SMJ19	5′GCAATACAGGGCTTCTTGGGGTGGGTG 3′
SMJ20	5′CACCCCTTTCCCACCCACCCCAA 3′
SMJ21	5′TTGGGGTGGGTGGGAAAGGGGTG 3′
SMJ22	5′TTGGGGTGGGTGGGAAACTAGTG 3′
SMJ23	5′TTGGGGTGGGTCTCAAAGGGGTG 3′
SMJ26	5′ACCCCTCGAACCCCCGCCCCTA 3′
SMJ27	5′TAGGGGCGGGGGTTCGAGGGGT 3′
SMJ28	5′ACCAACATCAGCTCCCTCAGCCCT 3′
SMJ29	5′AGGGCTGAGGGAGCTGATGTTGGT 3′
SMJ30	5′TCACACGCCCCCCTGAGTCCCT 3′
SMJ31	5′AGGGACTCAGGGGGGCGTGTGA 3′
SMJ32	5′TAGGGGCATTATTTCGAGGGGT 3′
SMJ33	5′AGGGACTCAGGGGGGCTTTTCA 3′
SLS25	5′ACCACAGTCCATGCCATCAC 3′
AP72	5'TCACGCGTCGACGCCCTTTCTTCACAGGTCAGT 3'
SS65	5′AGCTAACTGCATCTGTAGGTCGCGAATCAGTTCACTGCTTAA 3′
SD3.3	5′AGTGTGCGGAATGACTCAGG 3′
SD3.4	5′CCTGAGTCATTCCGCACACT 3′

Figure S1

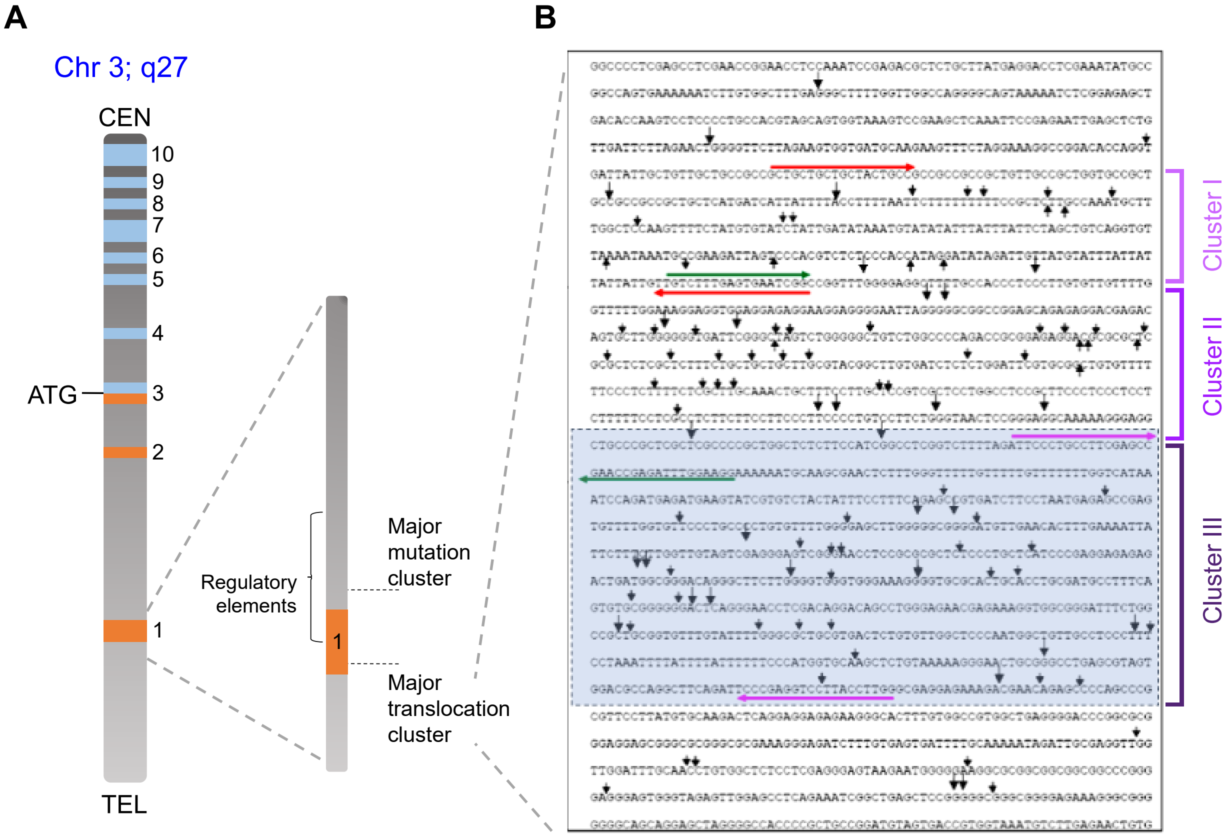

C

Inverted repeats (16)

Start	Stop	Length	Sequence
410	424	15	GCCAAATGCTTTGGC
737	750	14	GTCTGGCCCCAGAC
2130	2205	76	AACACAACCAAACCGATTTTTTAAAAAAA TGAAAAAAGACCCTCTGTGTTGTGTTCCC TTCTACGATGGTTGTGTT
2451	2513	63	CTTTTTTTAAAGTAAAGTGAGTGTCAGTA GATGTGTTTTTTACAGTCTGGATTTTAAA AAAAG
2871	2884	14	CTTTTTTAAAAAAG
3075	3090	16	TTGGAAGACTTTCCAA
3524	3536	13	ATTTTTGAAAAAT
3557	3651	95	AACATTTTTCCCCCAGAGATTTAAAAAAA ATAGGGGAGATAAAACTAACGGATAAAAA GGACTTTGAAGTTTATCAAGTAGGGAAGA AAAATGTT
3846	3860	15	TGGTGTAAAACACCA
4750	4763	14	GGAGGGCACCCTCC
5890	5909	20	CAGCCTTTTTGCAAAGGCTG
6091	6106	16	CTTGCAGGCTTGCAAG
7752	7769	18	GCTGCCGGGGCCGGCAGC
9765	9776	12	CCATGTACATGG
10645	10659	15	TGTGGTAAAACCACA
11444	11458	15	ATAGTGAAACACTAT

G quadruplex (8)

Start	Stop	Length	Sequence
1340	1373	34	GGGACAGGGCTTCTTGGGGTGGGTGGGAAAGGGG
1750	1777	28	GGGAGGAGCGGGCGCGGGCGCGAAAGGG
1935	1964	30	GGGGGCGGGCGGGGAGAAAGGGCGGGGGGG
6629	6656	28	GGGAAGGAGGGGAGGGGCGAGAAAGGGG
8541	8563	23	GGGTGCGGGGAGATTGGGATGGG
2333	2367	35	GGGTTGTGGGGTGGCGGGGGCCGGCGGGGAGGGGG
3022	3042	21	GGGGTGGCGGGCGGGGCGGGG
4118	4134	17	GGGAGGGTTGGGAGGGG

Direct repeats (3)

Start	Stop	Length	Sequence
7913	7935	23	AAAAAAAAAAAAAAAAAAAAAAA
8292	8340	49	TCTCTCTCTCTCTCTCTCTCTCTCTCTCTCTCTC TCTCTCTCTCTCTCT
11631	11652	22	AAAAACAAAAACAAAAACAAAA

Z DNA (2)

Start	Stop	Length	Sequence
5445	5457	13	GTGTGTGTGTGTG
7136	7146	11	GTGCGCGCGCA

Figure S2

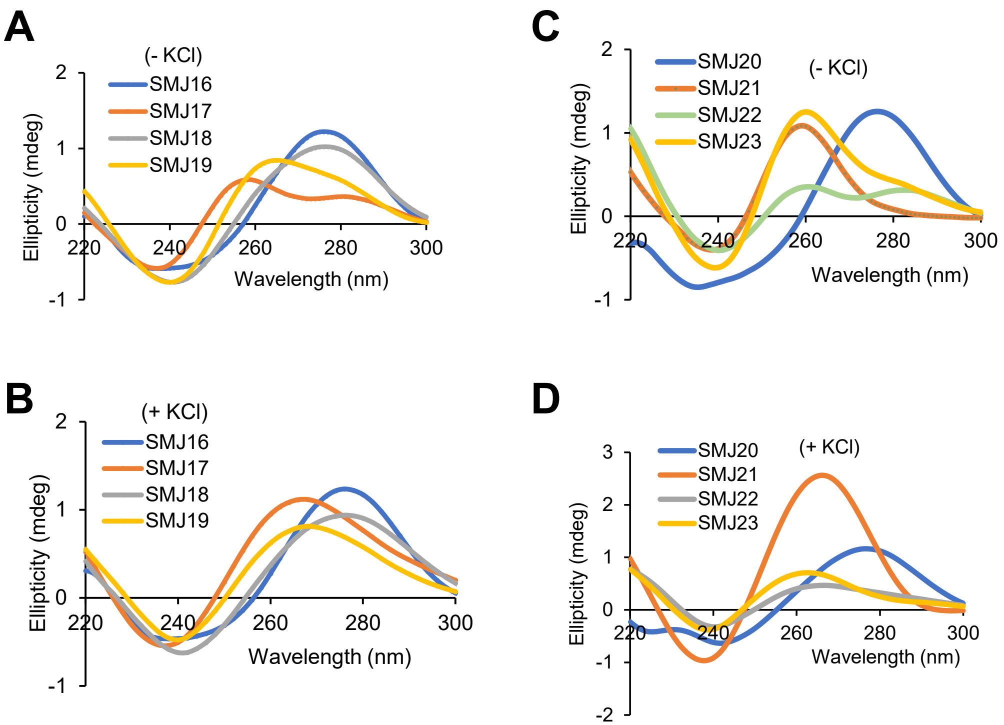
A
(- KCl)
SMJ16
SMJ17
SMJ18
SMJ19
Ellipticity (mdeg)
Wavelength (nm)
B
(+ KCl)
SMJ16
SMJ17
SMJ18
SMJ19
Ellipticity (mdeg)
Wavelength (nm)
C
(- KCl)
SMJ20
SMJ21
SMJ22
SMJ23
Ellipticity (mdeg)
Wavelength (nm)
D
(+ KCl)
SMJ20
SMJ21
SMJ22
SMJ23
Ellipticity (mdeg)
Wavelength (nm)

Figure S3

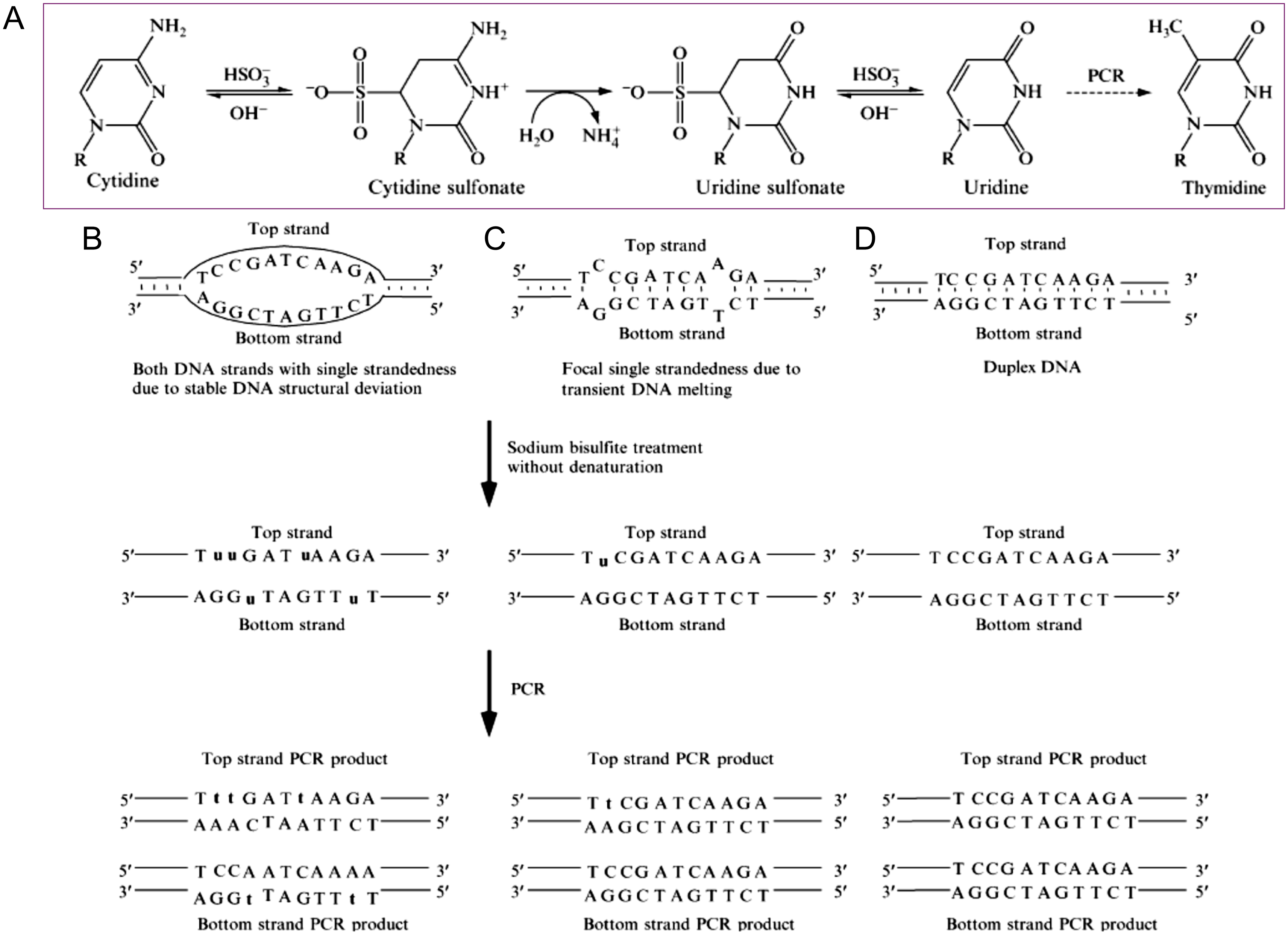

A
NH2
HSO3−
OH−
Cytidine
Cytidine sulfonate
H2O
NH4+
Uridine sulfonate
Uridine
PCR
H3C
Thymidine
B
Top strand
Bottom strand
Both DNA strands with single strandedness
due to stable DNA structural deviation
C
Focal single strandedness due to
transient DNA melting
D
Duplex DNA
Sodium bisulfite treatment
without denaturation
PCR
Top strand PCR product
Bottom strand PCR product

Figure S4

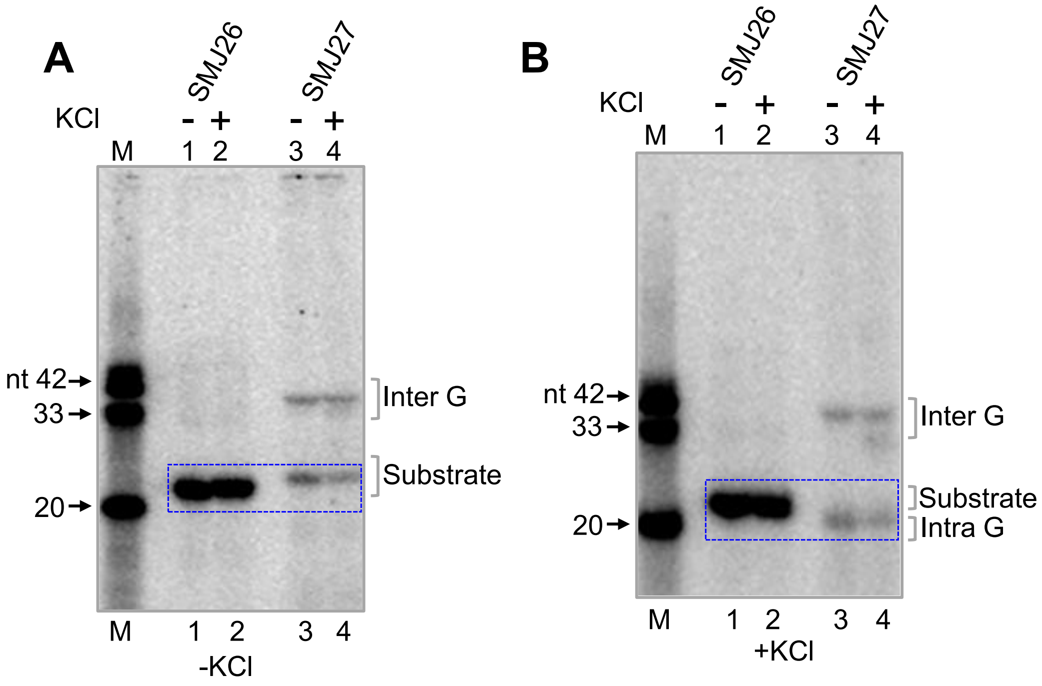
A
SMJ26
SMJ27
KCl
- + - +
M 1 2 3 4
nt 42
33
20
Inter G
Substrate
M 1 2 3 4
-KCl
B
SMJ26
SMJ27
KCl
- + - +
M 1 2 3 4
nt 42
33
20
Inter G
Substrate
Intra G
M 1 2 3 4
+KCl

Figure S5

A

SMJ26 5′ ACCCCTCGAACCCCCGCCCCTACA 3′

SMJ27 5′ TGTAGGGGCGGGGGTTCGAGGGGT 3′

B

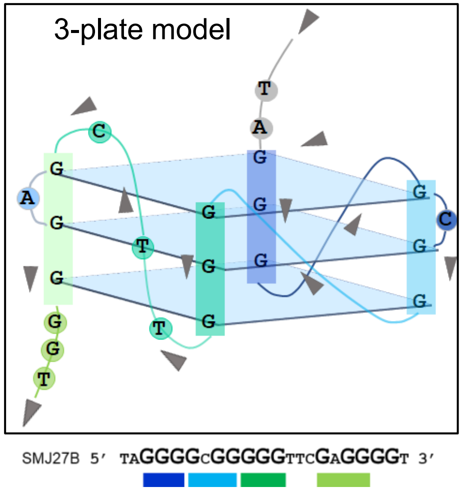

C

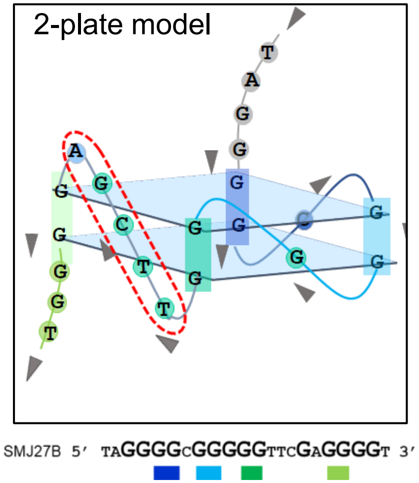

Supplement: Supplemental Figures [file mmc2.pdf]
